# Ca^2+^ inactivation of the mammalian ryanodine receptor type 1 in a lipidic environment revealed by cryo-EM

**DOI:** 10.7554/eLife.75568

**Published:** 2022-03-08

**Authors:** Ashok R Nayak, Montserrat Samsó

**Affiliations:** 1 Department of Physiology and Biophysics, Virginia Commonwealth University Richmond United States; Columbia University United States; The University of Texas at Austin United States

**Keywords:** rabbit, ryanodine receptor, inactivation, calcium, excitation-contraction coupling, cryo-electron microscopy, Other

## Abstract

Activation of the intracellular Ca^2+^ channel ryanodine receptor (RyR) triggers a cytosolic Ca^2+^ surge, while elevated cytosolic Ca^2+^ inhibits the channel in a negative feedback mechanism. Cryogenic electron microscopy of rabbit RyR1 embedded in nanodiscs under partially inactivating Ca^2+^ conditions revealed an open and a closed-inactivated conformation. Ca^2+^ binding to the high-affinity site engages the central and C-terminal domains into a block, which pries the S6 four-helix bundle open. Further rotation of this block pushes S6 toward the central axis, closing (inactivating) the channel. Main characteristics of the Ca^2+^-inactivated conformation are downward conformation of the cytoplasmic assembly and tightly knit subunit interface contributed by a fully occupied Ca^2+^ activation site, two inter-subunit resolved lipids, and two salt bridges between the EF hand domain and the S2–S3 loop validated by disease-causing mutations. The structural insight illustrates the prior Ca^2+^ activation prerequisite for Ca^2+^ inactivation and provides for a seamless transition from inactivated to closed conformations.

## Introduction

Ryanodine receptors (RyRs) are complex intracellular, multidomain Ca^2+^ channels that generate Ca^2+^ transients in cells of higher metazoans. They are pivotal for the contraction of skeletal and cardiac muscles (Flucher and Franzini-Armstrong, 1996; Meissner, 2017; Ríos, 2018), supporting a transient rise in cytosolic Ca^2+^ from its resting level of ~100 nM, which is driven by a more than 1000-fold Ca^2+^ concentration gradient across the membrane of the endoplasmic reticulum (sarcoplasmic reticulum in muscle [SR]) (Ziman et al., 2010). RyRs also play a role in neuron excitability (Albrecht et al., 2001; Arias-Cavieres et al., 2018; Bouchard et al., 2003) and a myriad of other Ca^2+^-dependent pathways such as differentiation, survival, and apoptosis (Bagur and Hajnóczky, 2017; Tu et al., 2016). Dysregulation of the channel leads to several life-threatening diseases such as malignant hyperthermia, central core disease, sudden cardiac death, and lethal fetal akinesia deformation sequence syndrome (Alkhunaizi et al., 2019; McCarthy et al., 2000; Priori et al., 2001; Tiso et al., 2001; Treves et al., 2008).

The high-conductance RyR channel is strongly regulated, with cytosolic Ca^2+^ concentration having a biphasic effect on the probability of channel opening. Notably, both intracellular Ca^2+^ channels, RyRs and inositol P_3_ receptors (IP_3_Rs), display a bell-shaped curve of Ca^2+^ dependence (Bezprozvanny et al., 1991; Meissner, 2017; Meissner et al., 1986), characteristic of dual regulation by cytosolic Ca^2+^. In RyR, the ascendant branch of Ca^2+^ dependence with half-maximal concentration (*K_a_*) of 1–5 μM (Laver, 2018; Laver and Lamb, 1998) is mediated by a high-affinity cytosolic Ca^2+^ site (des Georges et al., 2016). Ca^2+^ concentrations above 100 μM, reached in the tight nanodomain surrounding the RyR upon its opening (Langer and Peskoff, 1996), inhibit the channel (Chen et al., 1997; Xu and Meissner, 1998; Yamaguchi, 2020), which rapidly enters a refractory period that has been proposed to prevent depletion of the SR Ca^2+^(Bers, 2002; Ríos et al., 2008). Out of the three isoforms, the ‘skeletal muscle’ RyR1 isoform studied here is the most sensitive to Ca^2+^ inactivation (Meissner, 2017), where inactivation-impairing mutations are known to cause malignant hyperthermia (Gomez et al., 2016). While the inactivation of RyR by Ca^2+^ has been characterized at the functional level, its underlying mechanism is unknown. Outstanding questions are whether high Ca^2+^ induces an allosteric change in RyR that dampens the affinity of its activation site for Ca^2+^ and whether the Ca^2+^-inactivated conformation is similar or distinct with respect to the closed conformation obtained in the absence of Ca^2+^.

Here, cryogenic electron microscopy (cryo-EM) and single-particle 3D reconstruction were applied to rabbit RyR1 embedded in nanodiscs under conditions of partial Ca^2+^ inactivation. Classification of two independent cryo-EM datasets revealed, in both cases, the coexistence of closed and open conformations, in agreement with functional experiments performed on the same channels using tritiated ryanodine binding. In the closed (inactivated) conformation, the resolution of the cryo-EM maps of nanodisc-embedded RyR1 enabled visualization of two lipids buried in a pocket of the transmembrane domain (TMD). To our knowledge, this is the first time that lipid is visualized in direct contact with the RyR. The open state, obtained by classification of the same dataset, represents the first RyR1 open conformation achieved in the absence of any extra activator other than the physiological activators, Ca^2+^ and ATP. We also carried out a control 3D reconstruction of RyR1 under the same conditions except for the absence of Ca^2+^, which yielded a closed channel. A comparison of the closed conformations of RyR1 at high Ca^2+^ and of RyR1 in the absence of Ca^2+^ revealed unique features associated to Ca^2+^-inactivation. Both Ca^2+^-induced activation and inactivation can be explained by a unifying mechanism that involves conformational rearrangements within the central region of RyR1. Thus, the 3D structures of closed, open, and inactivated RyR1 embedded in lipidic nanodisc provide a mechanistic framework to understand the biphasic response of RyR1 to Ca^2+^. In addition, two inter-subunit salt bridges appear to mediate the Ca^2+^-inactivated structural rearrangement, a finding supported by disease-causing mutations hindering such interactions and known to impair Ca^2+^-induced inactivation of the RyR1.

## Results

### Experimental design and functional validation of RyR1

Experimental conditions were fine-tuned to obtain the inactivated state. The channels were prepared in 2 mM free Ca^2+^, a concentration that inactivates the channel. As RyR1 is constitutively bound to the sensitizing ATP in the muscle cell (Kushmerick et al., 1992; Meissner et al., 1986; Xu et al., 1996), we included its non-hydrolyzable form, AMP-PCP (ACP), for its structural determination. In order to visualize the direct effect of Ca^2+^ on RyR1’s conformation, FKBP12, a stabilizer of the cytoplasmic domain, was excluded. To determine the structure of RyR1 in a detergent-free, membrane-embedded natural state, RyR1 was reconstituted into membrane scaffold protein (MSP) 1E3D1 nanodiscs in the presence of phosphatidylcholine, an abundant phospholipid in membrane fraction preparations. As a control, we carried out cryo-EM and 3D reconstruction of RyR1 using identical buffer conditions and saturating ACP, and substituted Ca^2+^ by 1 mM EGTA plus 1 mM EDTA (dataset denominated RyR1-ACP/EGTA). The channels were also reconstituted into nanodiscs in the presence of phosphatidylcholine.

**Figure 1.**
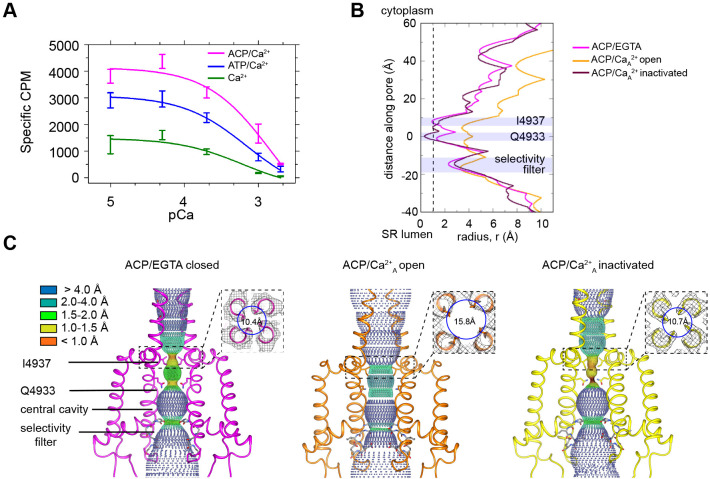
RyR1 at high Ca^2+^ concentration exhibits a mixture of open and inactivated conformations. (**A**) Ryanodine binding of rabbit skeletal sarcoplasmic reticulum (SR) microsomes showing the Ca^2+^-induced inactivation of RyR1; activators ATP or ACP (2 mM) increased channel open probability by 3- and 4.5-fold, respectively. Ca^2+^ concentrations of 100 μM to 2 mM progressively decrease the probability of opening (P_o_). Mean specific [^3^H]-ryanodine binding ± SEM from four independent experiments. (**B**) Pore profile of RyR1-ACP/EGTA, and of open and inactivated conformations in the RyR1-ACP/Ca^2+^_A_ dataset calculated with the program HOLE (Smart et al., 1993). The position of relevant landmarks is indicated with violet shadowing. Radius corresponding to a dehydrated Ca^2+^ ion shown with a dashed line. (**C**) Dotted surfaces of RyR1 ion permeation pathway in closed, open, and inactivated conformations, color-coded according to pore radius. Overlaid coordinates of the S5, S6, pore helices, and selectivity filter are shown for two diagonal protomers. Insets: cytoplasmic views of the Ile4937 constriction and corresponding pore diameter measured at the Cα backbone. Similar results were obtained for RyR1-ACP/Ca^2+^_B_ (Figure 1—figure supplement 5).

The Ca^2+^-induced activity profile of rabbit RyR1 in SR membranes was determined using the tritiated ryanodine binding assay, which reflects the probability of channel opening. The assay indicated RyR1 channel inactivation above 0.1 mM Ca^2+^, with an IC_50_ of 0.6 mM (Figure 1A). The presence of 2 mM ATP increased the efficacy of Ca^2+^-induced activation by approximately threefold at peak activation with similar potency for Ca^2+^-induced inactivation (IC_50_ of 0.7 mM), in agreement with earlier results obtained in lipid bilayer (Laver et al., 1995). We replicated the ryanodine-binding experiments over the same Ca^2+^ concentration range with 2 mM ACP. Maximal ryanodine binding was ~1.5-fold higher in the presence of ACP compared to ATP, and in this case slightly higher Ca^2+^ concentration was necessary for the same degree of RyR1 inhibition, with an IC_50_ value for Ca^2+^ of 1.5 mM in the presence of ACP. Thus, under our experimental conditions of 2 mM free Ca^2+^, RyR1 inactivation was partial.

**Figure 1—figure supplement 1.**
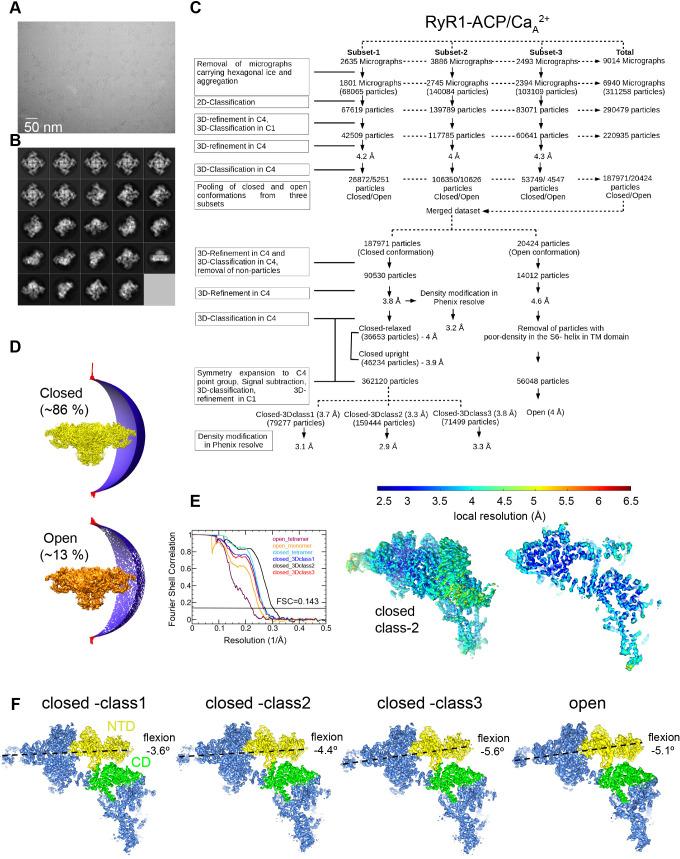
Image processing scheme for the RyR1-ACP/Ca^2+^_A_ dataset. (**A**) Representative micrograph collected on a Titan Krios at ×81,000 magnification with a K3 camera in counting mode. (**B**) Representative 2D class averages obtained by reference-free 2D classification in RELION. (**C**) Image processing workflow with tetrameric and single subunit-masked RyR1 particles in RELION. Overall resolution values obtained for the inactivated conformations after density modification in PHENIX.Resolve. (**D**) Euler angle distribution of the particles contributing to the RyR1-ACP/Ca^2+^ inactivated (3.8 Å resolution) and RyR1-ACP/Ca^2+^ open (4.6 Å resolution) maps. (**E**) Left: gold standard Fourier shell correlation prior to density modification. Right: local resolution of the symmetry-expanded maps calculated with ResMap. (**F**) Main 3D classes obtained from the focused 3D classification after symmetry expansion with NTD (N-terminal domain) and CD (Central domain) domains highlighted in yellow and green, respectively, with the pore axis on the right. Classification yielded one open conformation and three closed conformations that differ in the flexion angle of the cytoplasmic domain.

**Figure 1—figure supplement 2.**
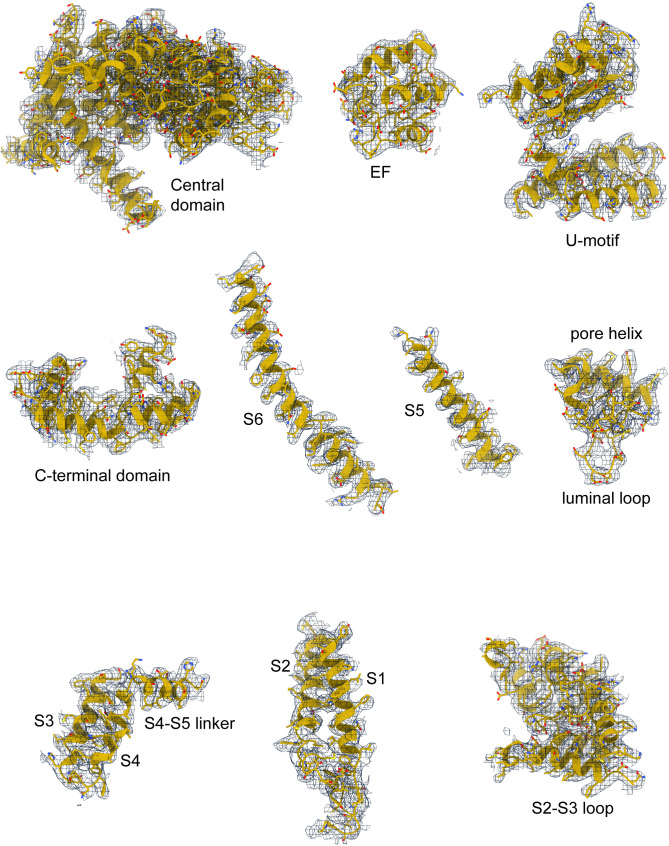
Correlation of RyR1-ACP/Ca^2+^_A_ inactivated model with the cryogenic electron microscopy (cryo-EM) density. Model quality for RyR1-ACP/Ca^2+^_A_ inactivated in the consensus cryo-EM map; domains in the central and transmembrane regions are shown.

**Figure 1—figure supplement 3.**
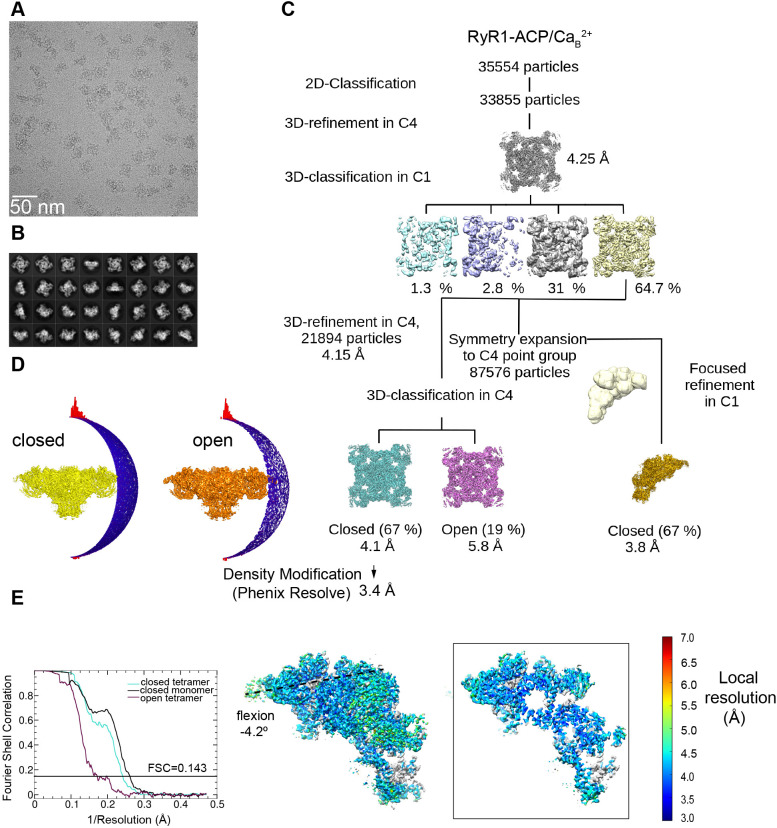
Image processing scheme for the RyR1-ACP/Ca^2+^_B_ dataset. (**A**) Representative micrograph collected on a Titan Krios at ×130,000 magnification with a K2 camera in super-resolution mode. (**B**) Representative 2D class averages obtained by reference-free 2D classification in RELION. Overall resolution values obtained for inactivated 3D class after a density modification in PHENIX.Resolve. (**C**) Workflow of image processing with tetramer and single subunit-masked particles carried out in RELION. (**D**) Euler angle distribution of the particles contributing to the RyR1-ACP/Ca^2+^_B_-inactivated and RyR1-ACP/Ca^2+^_B_ open maps at 4.1 Å and 5.8 Å resolution, respectively. (**E**) Left: gold standard Fourier shell correlation prior to density modification. Right: local resolution of the symmetry-expanded map calculated with ResMap with flexion angle of the cytoplasmic domain indicated.

**Figure 1—figure supplement 4.**
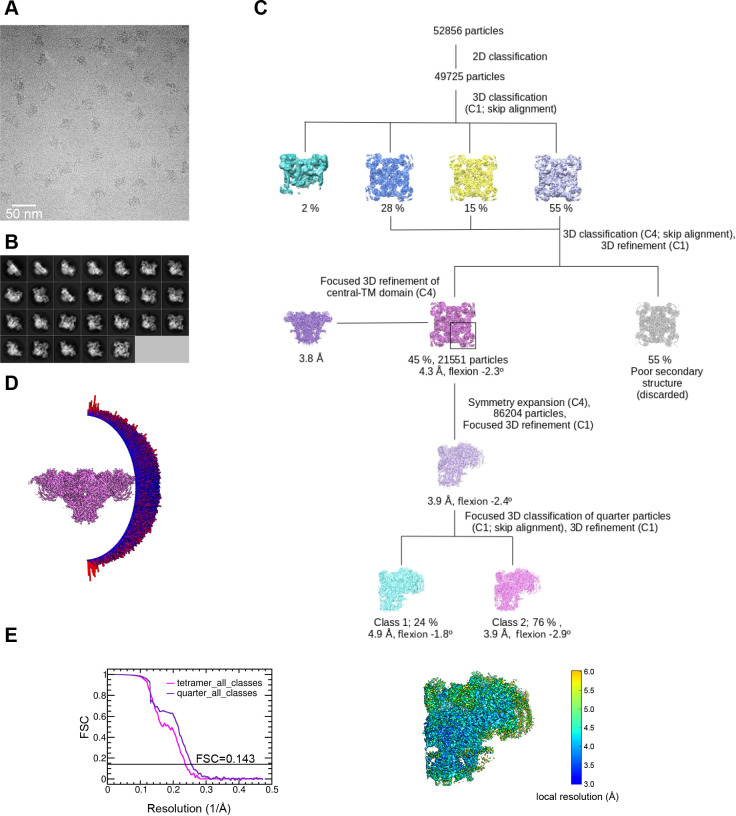
Image processing scheme for the RyR1-ACP/EGTA dataset. (**A**) Representative micrograph collected on a Titan Krios at ×130,000 magnification with a K2 camera in super-resolution mode. (**B**) Representative 2D class averages obtained by reference-free 2D classification in RELION. (**C**) Image processing workflow including 3D classification focused on RyR1’s quarter carried out in RELION. (**D**) Euler angle distribution of the particles contributing to the 4.3 Å resolution 3D map. (**E**) Left: gold standard Fourier shell correlation. Right: local resolution of the symmetry-expanded map calculated with ResMap.

**Figure 1—figure supplement 5.**
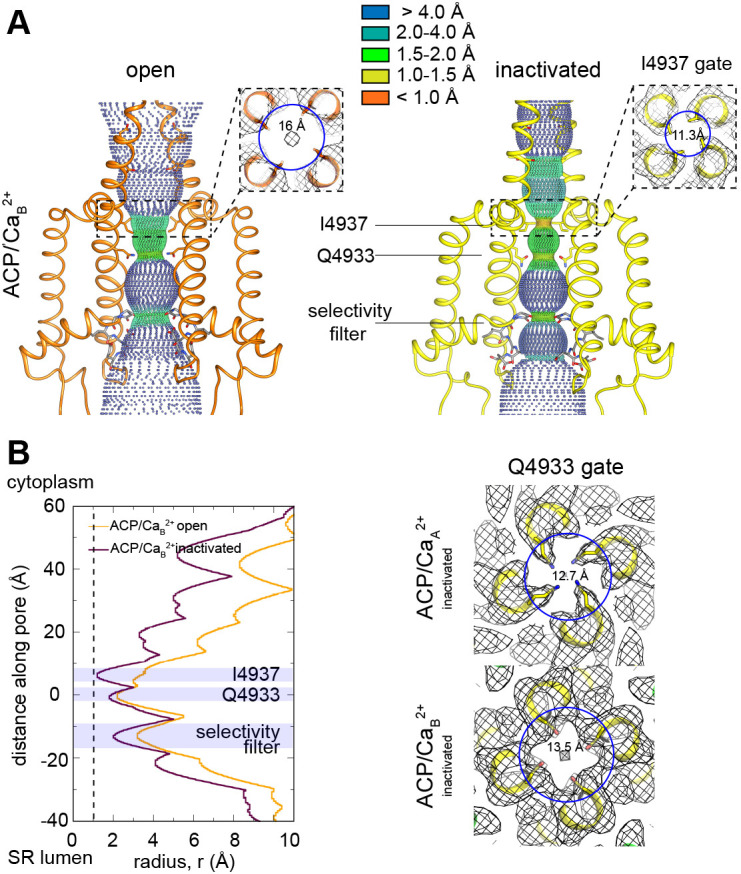
Pore conformation in the RyR1-ACP/Ca^2+^_B_ dataset. (**A**) Dotted surfaces of RyR1 ion-permeation pathway in open and inactivated conformations. Inset: fourfold cytoplasmic views of Ile4937 constriction and corresponding pore diameter (measured at Cα backbone) with respective cryogenic electron microscopy (cryo-EM) maps (mesh). (**B**) Left: pore profiles of inactivated and open conformations calculated with HOLE. Radius corresponding to a dehydrated Ca^2+^ ion is shown as a dotted line. Right: fourfold cytoplasmic views of the Gln4933 constriction in RyR1-ACP/Ca^2+^_A_ and RyR1-ACP/Ca^2+^_B_ pores with their respective cryo-EM maps.

### Classification of the high Ca^2+^ dataset reveals an *open* conformation and a *closed-inactivated* conformation

Cryo-EM of rabbit RyR1 embedded in nanodisc in the presence of 2 mM ACP and 2 mM free Ca^2+^ followed by single-particle analysis and 3D classification revealed two classes of particles, with their pore either fully open or fully closed. The findings, reproduced in two independent datasets (A and B), reflected the partial inhibition observed at 2 mM free Ca^2+^ (pCa 2.7) in the ryanodine-binding studies (Figure 1A). The 3D reconstructions of RyR1-ACP/Ca^2+^ open reported here are the first RyR1 open structures obtained in the presence of natural activators alone, possibly owing to the use of nanodiscs. The RyR1-ACP/Ca^2+^ reconstructions with a closed pore suggest a distinct conformation that we refer to as RyR1-ACP/Ca^2+^ inactivated henceforth, as further supported by our data.

A symmetry of C4 was imposed after ascertaining the true fourfold symmetry of the protein. The closed-pore (inactivated) channels were resolved to 3.8 Å and 4.1 Å resolution for the A and B datasets, respectively, which improved to 3.5 Å and 3.8 Å, respectively, using a phase improvement procedure (Terwilliger et al., 2020). Despite this improvement, we followed a conservative approach and used the non-density-modified maps for subsequent analysis. The open conformation represented a smaller fraction of the data (13 and 19% for the A and B datasets), which limited the resolution to 4.6 Å and 5.8 Å, respectively (Figure 1—figure supplement 1, Figure 1—figure supplement 2, Figure 1—figure supplement 3). The RyR1-ACP/Ca^2+^_A_ open subset improved to a resolution of 4.0 Å after a symmetry expansion step. Unless specified, the reported observations correspond to dataset A.

Classification of the control RyR1-ACP/EGTA dataset yielded a single class with a resolution of 4.3 Å (0.143 FSC gold standard), but further symmetry expansion and focused classification using a quadrant-shaped mask increased resolution to 3.9 Å (Figure 1—figure supplement 4). Despite the presence of the ACP activator, the 3D reconstruction of RyR1-ACP/EGTA yielded a closed state. Tables 1 and 2 summarize the cryo-EM data collection, single-particle image processing, model quality attributes, and database IDs for all datasets, and Table 3 compiles the main characteristics of the three conformations analyzed (closed, open, inactivated).

**Table 1.** Summary of cryogenic electron microscopy (cryo-EM) data collection and image processing parameters.

Dataset	RyR1ACP/EGTA _closed_	RyR1ACP/Ca2^2+^_A inactivated_	RyR1ACP/Ca2^2+^_A inactivated class1_	RyR1ACP/Ca2^2+^_A inactivated class2_	RyR1ACP/Ca2^2+^_A inactivated class3_	RyR1ACP/Ca2^2+^_A inactivated CDTM_	RyR1ACP/Ca2^2+^_A open_	RyR1ACP/Ca2^2+^_A open CDTM_	RyR1ACP/Ca2^2+^_B inactivated_	RyR1ACP/Ca2^2+^_B open_
**Data acquisition**										
Microscope/detector	Krios/K2	Krios/K3							Krios/K2	
Voltage (kV)	300	300							300	
Magnification	130,000	81,000							130,000	
Defocus range (μm)	–1.2 to –2.2	–1.25 to –2.5							–1.25 to –2.5	
Pixel size (Å) (calibrated)	1.06 (1.07)	1.08 (1.105)							1.06 (1.07)	
Total electron dose (e/Å^2^)	70	70							70	
Exposure time (s)	12	4.4							14	
Number of frames	60	50							50	
Total number of Micrographs	1959	10,002							1346	
**Image processing**										
Total number of particles selected	52,856	311,258							35,554	
Final number of particles	21,551	90,530	79,277	159,444	71,499	90,530	14,012	14,012	14,669	4160
Reconstruction symmetry	C4	C4	C1	C1	C1	C4	C4	C4	C4	C4
Map resolution, FSC (0.143) (Å) (symmetry expanded map)	4.3 (3.9)	3.8 (3.5)	3.7	3.3	3.8	3.5	4.6 (4.0)	4.4	4.1 (3.8)	5.8
Map sharpening B-factor (Å^2^) (symmetry expanded map)	–132	–150 (–144)	–125	–102	–123	–142	–168 (–135)	–144	–111(–89)	–148
EMDB ID	22616 22597	25828	25831	25830	25832	25828*	25829	25829*	25833	-

* Submitted as an additional map.

**Table 2.** Summary of model refinement and validation statistics.

Dataset	RyR1ACP-EGTA	RyR1ACP/Ca2^2+^A _inactivated_	RyR1ACP/Ca2^2+^ A _inactivated class1_	RyR1ACP/Ca2^2+^ A _inactivated class2_	RyR1ACP/Ca2^2+^ A _inactivated class3_	RyR1ACP/Ca2^2+^ A _open_
RMS deviation (bonds)	0.004	0.086	0.004	0.004	0.003	0.004
RMS deviation (angle)	0.919	0.812	0.893	0.788	0.848	0.766
**Ramachandran plot statistics (%)**						
Preferred	92.39	93.38	93.85	95.54	94.13	94.90
Allowed	7.45	6.57	5.97	4.38	5.75	5.07
Outliers	0.16	0.05	0.19	0.08	0.12	0.03
Clash-score	7.58	7.65	5.62	3.90	4.94	4.60
MolProbity score	1.89	1.85	1.71	1.49	1.65	1.59
PDB ID	7K0T	7TDG	7TDJ	7TDI	7TDK	7TDH

**Table 3.** Differences between closed, open, and inactivated conformations.

Conformation	Closed	Open	Inactivated
Pore	Closed	Open	Closed
Flexion angle (average)	–2.2°	–5.3°	–4.4°
In-plane rotation	0 (reference)	Counterclockwise	Counterclockwise
High affinity Ca^2+^ 900 site	Empty	Occupied	Occupied
Ca^2+^ bound to ACP	No	Yes	Yes
CD/CTD	Disconnected	Connected	Connected
Position of CD-C′	Down	Up/intermediate	Up
Position of CD-C′	Up	Down/intermediate	Down
EF hand/S2–S3 loop	Separated	Separated	Form two salt bridges
S6 TMD helix (4921-4928)	π	π wide-short	π narrow-long
Lipids in TMD crevice	Present	Absent	Present

TMD: transmembrane domain.

Pore analysis using the program HOLE (Figure 1B and C) of RyR1-ACP/EGTA indicated a closed pore with a radius constricting to 1 Å at the known hydrophobic gate Ile4937. In both RyR1-ACP/Ca^2+^ open A and B datasets, the hydrophobic gate as appraised by the pore profile (Figure 1B, Figure 1—figure supplement 5) and as measured at the S6 helix backbone C_α_ atoms (for more accurate measurement according to the lower resolution of the two datasets) (Figure 1C, Figure 1—figure supplement 5) had diameters of 15.8 Å and 16.0 Å, which indicates an open, Ca^2+^-permeable pore in both cases. Pore dimensions are comparable to the pore diameter of RyR1 open structures obtained in the presence of activators such as Ca^2+^/PCB95 or Ca^2+^/caffeine/ATP (16.7 Å; PDB ID: 5TAL; des Georges et al., 2016). The slightly narrower pore in our case could be attributed to the millimolar instead of submicromolar Ca^2+^ in our case and/or extra activators in addition to endogenous physiological activators in earlier open structures.

The reconstructions corresponding to RyR1-ACP/Ca^2+^ inactivated of the A and B datasets displayed a pore diameter at the Ile4937 gate of ~2 Å when considering the side chains, and 10.7 Å and 11.3 Å diameter, respectively, when measured at the backbone C_α_ atoms, making it impermeable to Ca^2+^ ions (Figure 1B and C, Figure 1—figure supplement 5). The position of the S6 backbone at Ile4937 is similar to our closed structure (*r* = 10.4 Å) and to RyR1-FKBP12/EGTA (*r* = 10.3 Å; PDB ID: 5TB0; des Georges et al., 2016). Interestingly, the channel pore was narrower than 1 Å at Gln4933 in RyR1-ACP/Ca^2+^_A_ inactivated (Figure 1B and C).

We previously established that the large square-shaped cytoplasmic shell of the RyR undergoes a conformational change upon opening. The periphery of its four quadrants tilts downward (toward the membrane), while its inner corner tilts away from it, rotating around a pivot point (Samsó et al., 2009). Such tilt can be quantified with the flexion angle, whereby negative angle (downward) correlates with opening, positive angle with closing, and absence of FKBP lowers the flexion angle of closed states (Steele and Samsó, 2019). In general, the approximate ranges of flexion angles are +1° to +2° for RyR1-FKBP12/EGTA closed, –1° to –3° for RyR1-EGTA closed, 0° to –3° for RyR1-FKBP12 ‘primed’ (with activating ligands and closed pore), and –1.5° to –5° for RyR1-Ca^2+^ open with or without FKBP12 (Iyer et al., 2020; Steele and Samsó, 2019). Here, RyR1-ACP/Ca^2+^ open channels have flexion angles of –5.1° and –4.8° for the A and B datasets, within the expected range. The consensus 3D reconstruction of RyR1-ACP/EGTA had flexion angle of –2.2°, and its two classes had flexion angles of - 1.8° (24%) and –2.9° (76%) (Figure 1—figure supplement 4). But unexpectedly for closed-pore channels, RyR1-ACP/Ca^2+^ inactivated datasets have a negative flexion angle (–4.6° and –4.2° for A and B datasets, respectively) (Figure 1—figure supplements 1 and 3). As there was an indication of variability in the cytoplasmic shell of RyR1-ACP/Ca^2+^_A_-inactivated dataset, we carried out fourfold symmetry expansion and focused classification for the monomer and obtained three subclasses, all showing downward motion: class 1 (22% of particles, 3.7 Å resolution, –3.6° flexion angle), class 2 (44% of particles, 3.3 Å resolution, –4.4° flexion angle), and class 3 (20% of particles, 3.8 Å resolution, –5.6° flexion angle) (Figure 1—figure supplement 1). Such pronounced downward flexion angles in a closed channel cannot be explained just by the lack of FKBP12 in our preparations. The distinct conformation of RyR1-ACP/Ca^2+^ inactivated, consisting of a closed pore and extreme-downward cytoplasmic assembly, prompted further analysis of the central region that joins the cytoplasmic and transmembrane domains.

### Different arrangement of the central region in Ca^2+^-inactivated, closed and open conformations

**Figure 2.**
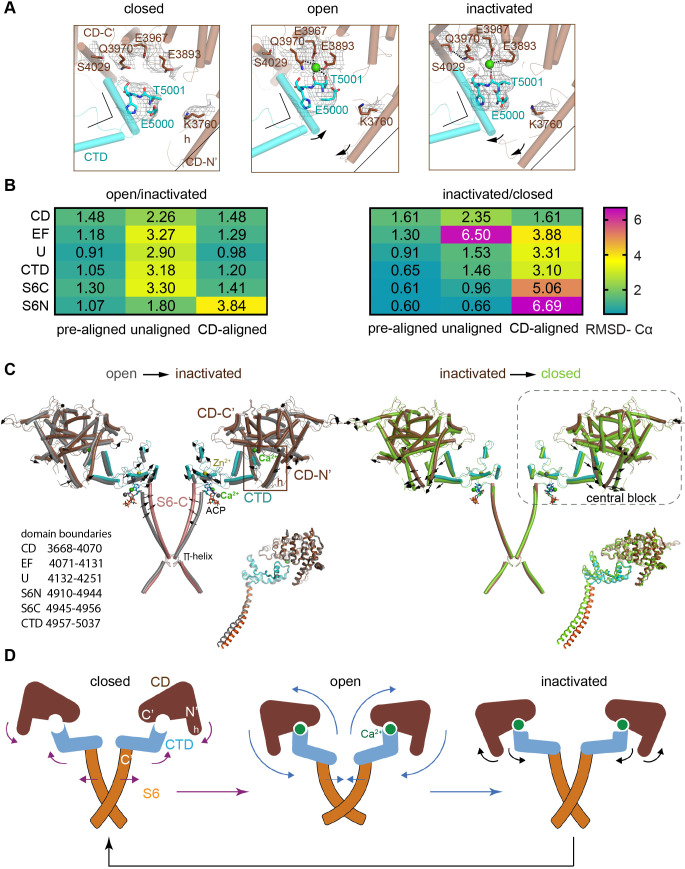
Inactivation of RyR1 involves out-of-plane rotation of the central block and rearrangement around the Ca^2+^ activation site. (**A**) The high-affinity Ca^2+^-binding site in the CD/CTD interface with density around the Ca^2+^ site contoured at 4σ. Contacts within 2.8 Å from Ca^2+^, as well as additional contact Gln3970-Ser4029 within 3.6 Å, are represented by dashed lines. Channel axis is on the left. During the transition from open to inactivated conformations, the CD/CTD block tilts around the Ca^2+^-binding site such that the protruding fourth helix of the CD (h) and connected CTD tilt inward, while the CD-C′ tilts upward and away from the sarcoplasmic reticulum (SR) membrane – with Gln3970 separating from Ca^2+^ by ~6 Å. Arrows and stationary reference lines illustrate the conformational changes undergone with respect to the panel on the left. The region represented relative to the channel is highlighted with a square in panel (**C**). See Figure 2—figure supplement 1 for the corresponding space-filling representation. (**B**) Heat map showing Cα-backbone root mean square deviation (RMSD) (in Å) between domain pairs from respective conformations after aligning them (pre-aligned), in their native conformation (unaligned), and after aligning the protomers through their respective CD (CD-aligned). The RMSD difference between unaligned and pre-aligned represents the change caused by domain relocation. (**C**) Overlaid structures of the CD-CTD-S6 domains in different conformations; only two protomers shown for clarity. Left: in the transition from RyR1-ACP/Ca^2+^ open (gray) to RyR1-ACP/Ca^2+^ inactivated (colored), the CD-CTD block, ‘connected’ by Ca^2+^ coordination, undergoes an out-of-plane rotation around the Ca^2+^-binding site that pushes S6C′ toward the pore axis, closing the channel. Right: in the transition from RyR1-ACP/Ca^2+^ inactivated (colored) to RyR1-ACP/EGTA (green), Ca^2+^ unbinding disconnects the CD from the CTD. Structures at the bottom right of each panel show the comparison of the CD-CTD-S6C′ of the central block after forcing superimposition of their respective CDs. Residue boundaries for relevant domains are specified. (**D**) Schematics of the conformational changes from RyR1-ACP/EGTA (closed), to RyR1-ACP/Ca^2+^ open, to RyR1-ACP/Ca^2+^ inactivated. Activation, where Ca^2+^ binds to the high-affinity site, is required prior to inactivation. The CD-protruding fourth helix (3753–3769) is indicated by ‘h.’ Colored arrows indicate the conformational change toward the following structure.

In RyR1-ACP/Ca^2+^ open, the high-affinity Ca^2+^-binding site was formed by Glu3967, Glu3893 (from the CD; residues 3668–4070), and Thr5001 (from the CTD (C-terminal domain); residues 4957–5037) as reported earlier (des Georges et al., 2016), and additionally Gln3970 in our case, which was visible up to 20σ. The Ca^2+^-induced reorientation of CTD with respect to CD and subsequent separation of S6 was obvious when compared to the RyR1-ACP/EGTA structure (Figure 2A, Figure 2—figure supplement 1), consistent with previous reports of Ca^2+^-induced activation (Bai et al., 2016; des Georges et al., 2016).

**Figure 2—figure supplement 1.**
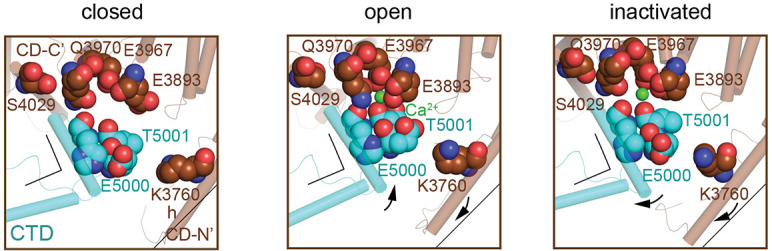
Reorganization of the high-affinity Ca^2+^-binding site at the CD/CTD interface under different conditions. Ca^2+^-binding site in the CD/CTD interface in different conformations with involved residues shown with spheres. Residues Glu3983, Glu3967, Gln3970 (CD), and Thr5001 (CTD), within 2.8 Å from Ca^2+^ in the open state, are shown. In going from RyR1-ACP/Ca^2+^ open to RyR1-ACP/Ca^2+^ inactivated, Gln3970 switches its interaction from Ca^2+^ to Ser4029 (interaction within 3.6 Å). Arrows and stationary reference lines illustrate the conformational changes undergone with respect to the panel on the left. The region represented relative to the channel is highlighted with a square in Figure 2C; fourfold axis is on the left.

**Figure 2—figure supplement 2.**
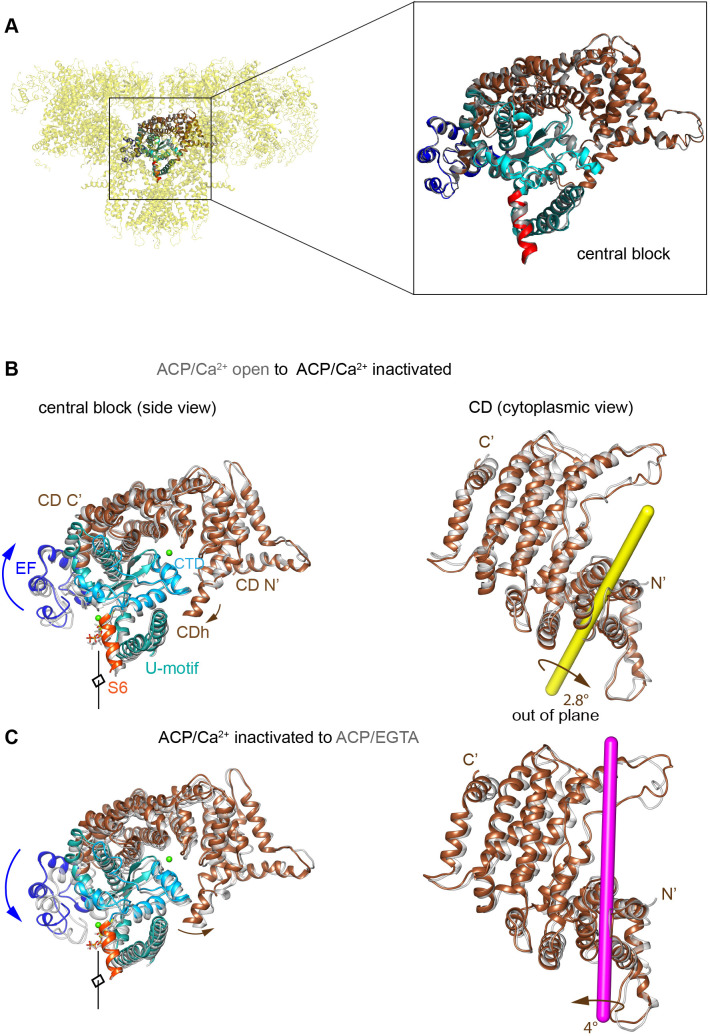
Rotation axes of the CD of RyR1 among the different conformational transitions. (**A**) Reproducibility of the conformation of the ‘central block’ of RyR1-ACP/Ca^2+^ inactivated from two independent datasets (A and B, superimposed), with a root mean square deviation (RMSD) of 0.95 Å over the Cα atoms. The central block comprises the CD, EF hand, U-motif, S6C′, and CTD. (**B**) Transition from RyR1-ACP/Ca^2+^ open (gray) to RyR1-ACP/Ca^2+^ inactivated (colored) involves an out-of-plane rotation of the CD around the yellow-colored axis. This results in inward tilt in CD-N′ (brown arrows) and upward shift/tilt of CD-C′ and connected EF hand domain (blue arrows). Two orthogonal views are shown, and the fourfold axis on the left panels, in front of the structure, is indicated. (**C**) Transition from RyR1-ACP/Ca^2+^ inactivated (colored) to RyR1-ACP/EGTA closed (gray) involves out-of-plane rotation of the CD of RyR1 around the magenta-colored axis.

**Figure 2—video 1.** Ca^2+^-induced transitions in activation and inactivation. During activation, Ca^2+^ coordination joins the CD and CTD-S6C′ region, separating the S6 four-helix bundle and opening the channel. High Ca^2+^-induced inactivation promotes out-of-plane rotation of the CD-CTD-S6C′ block. This pushes the S6 helices towards the pore axis, closing the channel. Only two protomers in diagonal are shown.

An outstanding question is how higher Ca^2+^ concentration may result in inactivation. Potential mechanisms are an additional allosteric change that dampens the affinity of the Ca^2+^ activation site, or that the high-affinity Ca^2+^ site remains occupied while an additional conformational change overcomes activation. Consistent with the second scenario, the high-affinity Ca^2+^-binding site in RyR1-ACP/Ca^2+^ inactivated remained fully occupied by Ca^2+^ up to 20σ, although with additional out-of-plane tilting of the CD and CTD around the Ca^2+^-binding site. The Ca^2+^ ion was coordinated by Glu3893, Glu3967 (CD), and Thr5001 (CTD) within contact distances of 2.8 Å, consistent with the open structures. However, on the CD-C' side, Gln3970 lost coordination to Ca^2+^, interacting with Ser4029 instead (Figure 2A, Figure 2—figure supplement 1). Mutations Q3970E/K in RyR1 are implicated in central core disease and equivalent RyR2 mutations in cardiac arrhythmia (Chirasani et al., 2019), which highlights the important Ca^2+^ sensing role for this residue.

To understand how the fully occupied high-affinity Ca^2+^-binding site (CD/CTD interface) led to a closed pore in RyR1-ACP/Ca^2+^ inactivated, we looked for differences between RyR1-ACP/Ca^2+^ open and RyR1-ACP/Ca^2+^ inactivated in the domains spanning from the CD/CTD interface to the pore. Specifically, we focused on domains around the high-affinity Ca^2+^-binding site (CD, EF hand, U-motif, CTD, and the C′ section of S6 or S6C′; domain boundaries indicated in Figure 2), which we term ‘central block.’ A comparison was done for dataset A after confirming similarity for the central block of 0.95 Å Cα root mean square deviation (RMSD) for the encompassed 672 resolved residues, between inactivated A and B datasets (Figure 2—figure supplement 2A). The RMSD (Cα backbone) between individual domains from RyR1-ACP/Ca^2+^ inactivated versus open was below 1.5 Å for all comparisons when the domains were pre-aligned pairwise (Figure 2B, Figure 2—figure supplement 2B and C), indicating little conformational change. The N′ section of S6 was also included in the comparisons. Without pre-alignment, RMSD was higher (Figure 2B), indicating domain repositioning, the degree of which was estimated by subtracting the pre-aligned from the unaligned RMSDs. This yielded an average shift of ~2 Å per domain in going from RyR1-ACP/Ca^2+^ open to RyR1-ACP/Ca^2+^ inactivated, except for S6N’ where the shift was ~0.7 Å (Figure 2B). Comparing the same domains between RyR1-ACP/Ca^2+^ inactivated and RyR1-ACP/EGTA (closed) yielded smaller repositioning with average shifts of ~1 Å, reflecting similarity in the domain’s locations, with the exception of the EF hand domain that undergoes an ~5 Å repositioning (Figure 2B, Figure 2—figure supplement 2C).

When the central blocks were pre-aligned as a unit using their respective CD domains, and the individual domains were compared pairwise (CD-aligned; Figure 2—figure supplement 2A), there was a good overlap between RyR1-ACP/Ca^2+^ open and RyR1-ACP/Ca^2+^ inactivated (RMSD below 1.5 Å) except for S6N′ (3.8 Å), revealing the bend in the middle of S6 upon opening. In contrast, the CD-aligned RMSD of RyR1-ACP/Ca^2+^ inactivated vs. RyR1-ACP/EGTA closed was higher, between 3 and 4 Å for the EF hands, U-motif, and CTD, and increases toward the C-terminus, 5 Å for S6C′ and almost 7 Å for S6N′ (Figure 2B, ‘CD-aligned’). This indicated that domains CD, EF hands, U-motif, S6C′, and CTD relocated together, and that the central block became more compact in the presence of Ca^2+^ acting at the CD/CTD interface. Taken together, these results imply that conformational changes from CD are transmitted to S6 only in the presence of Ca^2+^ (Figure 2C and D).

We analyzed how the central block may evolve from RyR1-ACP/EGTA (closed), to RyR1-ACP/Ca^2+^ open, to RyR1-ACP/Ca^2+^ inactivated. Upon Ca^2+^-induced opening, ‘engagement’ of CD and CTD by Ca^2+^ tilts the CD out of plane (such that helix h tilts 3° inwards), while the CTD tilts up and outwards. This pulls the S6 helices (which are directly connected to the CTD) away from the pore axis, opening the ion pathway (Figure 2C and D). To reach the inactivated state, the central block tilts further (see rotation axis in Figure 2—figure supplement 2B) in a movement similar to pushing down the levers of a winged corkscrew, which pushes each S6 helix 2.5 Å toward the pore axis, closing the channel. Alpha helices within the CTD act as a lever coupling this out-of-plane rotation to S6C′ and as described further below, ATP reinforces the connection between CTD-S6C′ in the presence of Ca^2+^. These conformational changes involved in activation, inactivation, and transition from the inactivated to the closed state are shown in the morph among conformations in Figure 2—video 1 and illustrated in the schematics in Figure 2D.

### Formation of salt bridges between EF hand domain and S2–S3 loop of the neighboring subunit in the inactivated state

**Figure 3.**
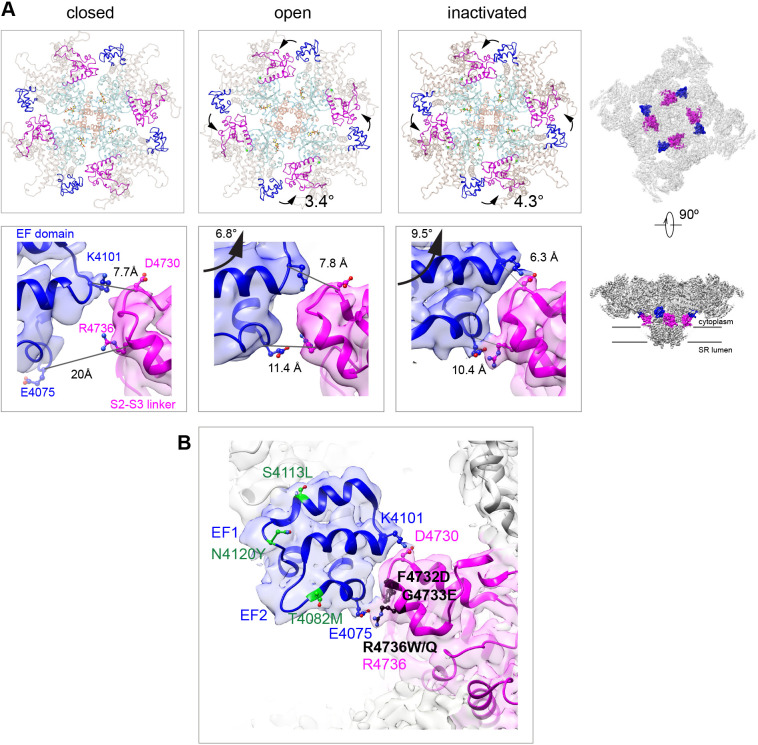
Two salt bridges between the EF hands and the S2–S3 loop are determinant for the inactivated state. (**A**) Cytoplasmic sarcoplasmic reticulum (SR) view (top row) and side view (bottom row) highlighting the EF hand domain (blue) and S2–S3 loop (magenta) at the subunit interface. Counterclockwise in-plane and ascending out-of-plane rotations of the central region in the transition from RyR1-ACP/EGTA (closed) to RyR1-ACP/Ca^2+^ open brings the two domains closer together. Further rotation of the CD (tan color in top row) and its protruding EF hand domain in RyR1-ACP/Ca^2+^ inactivated brings the two domains in contact. The inter Cα-Cα contact distances illustrate the progressive approximation of the two domains. Two salt bridges, Lys4101-Asp4730 and Glu4075-Arg4736, form in the RyR1-ACP/Ca^2+^-inactivated conformation. The location of the domains in the context of RyR1 is shown on the right panels. (**B**) MH/CCD mutation sites in the interface between the EF hand domain and S2–S3 loop that abolish Ca^2+^-dependent inactivation (black) versus MH/CCD mutations without effect on inactivation (green). The two EF loops are indicated (EF1, EF2). Residues forming the salt bridges are labeled according to domain color. R4736, directly forming the salt bridge, is susceptible to MH mutation.

The EF hand domain of RyR1, a candidate Ca^2+^ regulation site (Du et al., 1998; Gomez et al., 2016; Gomez and Yamaguchi, 2014; Xiong et al., 1998), did not show significant changes, with maximum Cα pre-aligned RMSD of 1.5 Å among the different states analyzed (closed, open, inactivated). The EF hand loops (residues 4081–4090, 4116–4123) were empty in our high Ca^2+^ conditions, which is consistent with the low affinity of Ca^2+^ to this site (K_d_ 3.7 mM, Xiong et al., 1998). Nonetheless, the entire EF hand domain, which protrudes from the CD-C′, repositions noticeably during activation, with a 3.4° counterclockwise in-plane rotation (as seen from the cytoplasmic side), and 6.8° upward out-of-plane rotation. This movement brings the EF hand domain in closer proximity to the S2–S3 loop (cytoplasmic loop between S2 and S3 TMD helices; residues 4664–4786) (Figure 3). With inactivation, an additional 1° counterclockwise rotation and 2.7° upward out-of-plane rotations define a physical limit to the counterclockwise motion, forming two salt bridges at this inter-subunit interface: Glu4075-Arg4736 and Lys4101-Asp4730, with their side chains within 3.5 Å (Figure 3A). This interaction is critical to support inactivation, as demonstrated by the fact that MH/CCD mutations facing this interface F4732D, G4733E, and R4736W/Q, with the latter including the Arg directly involved in the salt bridge (Figure 3B), greatly reduced channel inactivation (Gomez et al., 2016). On the other hand, MH/CCD mutations T4082M, S4113L, and N4120Y, in regions of the EF hand domain away from the interface (Figure 3B), did not affect RyR1 inactivation (Gomez et al., 2016), serving as a negative control for this hypothesis.

### Ca^2+^ binds to the ATP-binding pocket

**Figure 4.**
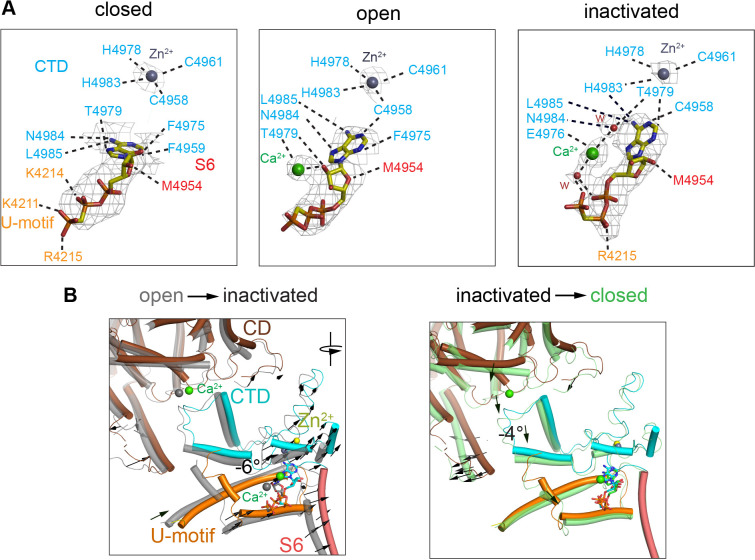
Changes in the interaction network of the nucleotide and complexed Ca^2+^. The ATP-binding pocket is formed by the U-motif, CTD, and S6C′. (**A**) cryo-EM densities (gray mesh) of ACP in the different states represented at map significance values (root mean square deviation [RMSD] σ) above 12, 7, and 7, respectively. Residues within 4 Å of either ACP, Ca^2+^, or Zn^2+^ (from the CTD zinc finger) are color-coded according to domain. Notably, an interaction between His4983 (CTD) and ACP is only observed in the inactivated state. Fitted water densities (w) are represented in red. (**B**) Left: ACP goes deeper into the ATP-binding pocket in RyR1-ACP/Ca^2+^ inactivated (colored structure, blue ACP) as compared to the open state (gray structure, red ACP) due to a 6° rotation (see arrows). Right: absence Ca^2+^ in the closed state (green) causes release of the CTD from the CD, resulting in a 4° tilt of the CTD and U-motif (see arrows). Additional reorganization allows closer interaction between ACP and U-motif in the closed state.

The ATP-binding site showed full occupancy in the three conformations – open, closed, and inactivated. Under closed-state conditions, ACP bound to the pocket formed by the U-motif, S6C′, and CTD (Figure 4A, ‘closed’), in the same position as ATP (des Georges et al., 2016), with a high map significance (7σ). An additional elongated non-protein density was associated to ACP in the RyR1-ACP/Ca^2+^ open and inactivated maps. This distinct density has high map significance (15σ) (Figure 4A, Figure 4—figure supplement 1), suggestive of a putative Ca^2+^ ion. Well-resolved local density in RyR1-ACP/Ca^2+^_A_-inactivated class 1 map allowed tentative modeling of two waters on either side of the putative Ca^2+^ that connect on either side to ACP’s γ-phosphate and Thr4979 (CTD) (Figure 4A, ‘inactivated’), suggesting potential coordination through Ca^2+^’s first layer of hydration. Analysis of protein-ligand interactions based on the atomic coordinates (Laskowski and Swindells, 2011) showed an increased network of electrostatic and hydrophobic interactions, and three predicted hydrogen bonds, that the inactivated conformation gained with respect to closed conformation (Figure 4A). Thus, the nucleotide nestled deeper into the cavity in going from closed to open, and then to inactivated, increasing connectivity between S6C′ and CTD and reducing connectivity to the U-motif (Figure 4B). Interestingly, ACP in RyR1-ACP/Ca^2+^_A_ inactivated acquired an interaction with the backbone carbonyl of His4983, a residue that participates in the C2H2 zinc motif that is central to the CTD (Figure 4). Based on the higher map significance of the putative Ca^2+^ ion in our cryo-EM maps obtained at higher Ca^2+^ concentration (Figure 4—figure supplement 1) and the enhanced inactivation when ATP is present (Sitsapesan and Williams, 2000), the interface between the nucleotide, the CTD, and S6 could be thought of as an atypical low-affinity Ca^2+^ inactivation site. The picture is bound to be more complex as presumably Mg^2+^ present in the cytoplasm would also bind to ATP. Thus, under physiological conditions of high local Ca^2+^, competition between the two divalent cations for ATP may take place.

**Figure 4—figure supplement 1.**
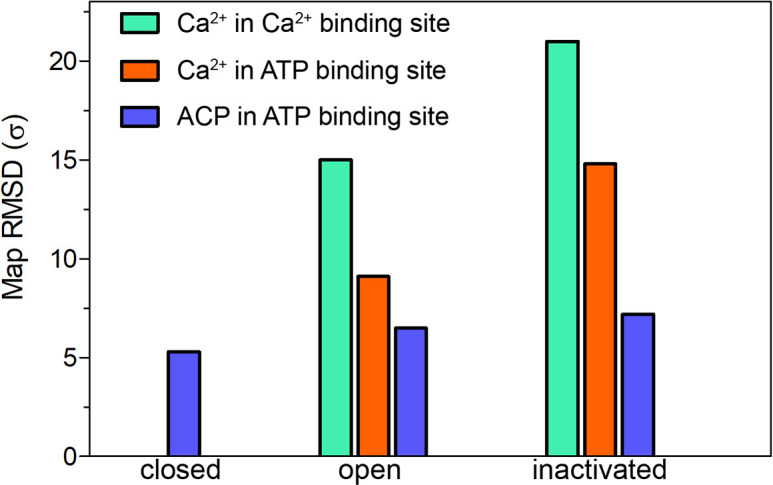
Map significance of the ACP and putative Ca^2+^ densities under different conditions. Root mean square deviation (RMSD σ) values of the densities attributed to Ca^2+^ and ACP in the cryogenic electron microscopy (cryo-EM) maps of RyR1-ACP/EGTA closed, RyR1-ACP/Ca^2+^ open, and RyR1-ACP/Ca^2+^ inactivated.

### Protein-lipid interactions within the nanodisc environment

**Figure 5.**
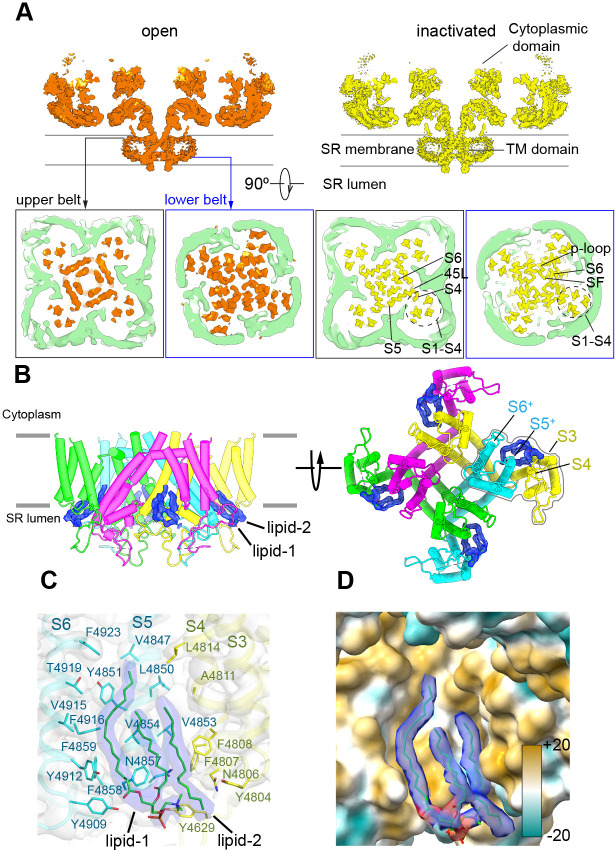
The nanodisc environment and visualization of lipids in a crevice of the transmembrane domain (TMD). (**A**) Top: central slice of the side view of the RyR1-ACP/Ca^2+^_A_ open and RyR1-ACP/Ca^2+^_A_-inactivated cryogenic electron microscopy cryo-EM maps highlighting the density corresponding to the upper and lower nanodisc belts. Bottom: corresponding views seen from the cytoplasmic direction. For clarity, the nanodisc density (green) was extracted and low-pass filtered to 7 Å resolution. The top belt of the nanodisc expands slightly to accommodate the conformational change; see also Figure 5—video 1. (**B**) Side and luminal views of the TMD of RyR1-ACP/Ca^2+^_A_ inactivated with putative lipid densities shown in blue. (**C**) Lipid-binding pocket of RyR1-ACP/Ca^2+^_A_ inactivated lined by lipophilic amino acids from S3 and S4 of the voltage sensor-like domain (S1–S4; yellow) and core helices S5 and S6 (cyan) from two different protomers. Amino acids within 5 Å from the lipids are shown (sticks) with their corresponding side chain densities. Densities corresponding to the lipids contoured at 8σ (in blue) are modeled as a PC (16:0-11:0) for lipid 1 and a 16C acyl chain for lipid 2. (**D**) Molecular lipophilicity potential of the surface lining the crevice, ranging from hydrophilic (cyan) to hydrophobic (golden). The hydrophobic tails of the lipids are shown in blue, and the negative electrostatic surface potential of the polar lipid head is shown in red.

To provide a more physiological environment to the TMD and avoid the presence of detergents, we embedded the protein in scaffold protein MSP1E3D1 that assembled into nanodiscs. The reconstituting lipid was phosphatidylcholine. Refinement focused on the CD-TMD region of RyR1-ACP/Ca^2+^_A_ inactivated and open, resulting in reconstructions with 3.5 Å and 4.4 Å resolution, respectively, yielded a visible electronfor the nanodisc. Two molecules of the MSP1D1E3 scaffold protein wrapped closely around the TMD in a double-belt arrangement (Figure 5A). The larger top belt adopts a quatrefoil shape, with one voltage sensor (S1–S4) in each lobe, while the lower belt is rounder and smaller, following the tapered TMD. The larger footprint of the top half of RyR1’s TMD, reflected in the surrounding nanodisc, appears to correlate with the curvature of the membrane around RyR1 observed by electron tomography in its native membrane (Chen and Kudryashev, 2020; Renken et al., 2009). Even considering the tight fit between the nanodisc and RyR1’s TMD, conformational changes were unhindered and the channel opened within the nanodisc environment. The slight expansion of the top belt of the nanodisc at the level of the ion gate upon opening (Figure 5—video 1) reveals a certain degree of plasticity of the scaffold protein.

**Figure 5—figure supplement 1.**
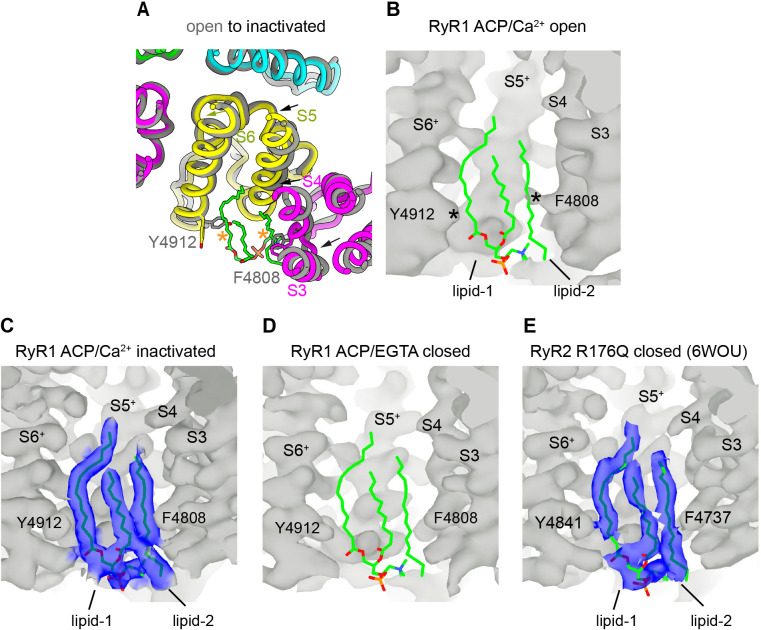
Lipid-binding pocket lined by the S3/S4 and S5^+^/S6^+^ helices in inactivated and closed structures of RyR suggests a conserved functional site. (**A**) Cytoplasmic fourfold view of part of the transmembrane domain (TMD) showing the movement of the transmembrane helices S3, S4, and S5 (arrows) into the lipid pocket while transitioning from RyR1-ACP/Ca^2+^ open to inactivated conformations. No ordered lipids were observed in the open channel. Each subunit is displayed with a different color. (**B**) Side view of the lipophilic pocket in RyR1-ACP/Ca^2+^ open with overlaid lipid backbone extracted from the RyR1-ACP/Ca^2+^-inactivated conformation, showing expected steric hindrance of lipid 1 and lipid 2 with S6 and S4, respectively, as indicated with asterisks. See also Figure 6A, bottom panels. (**C**) Side view of RyR1-ACP/Ca^2+^ inactivated with resolved lipids. (**D**) Side view of the lipophilic pocket in RyR1-ACP/EGTA, with lower resolution but foreseeable lack of steric hindrance between protein and lipids. Overlaid lipid backbones are extracted from the inactivated conformation. (**E**) Side view of the lipophilic pocket in RyR2 closed at 3.27 Å resolution (PDB ID: 6WOU; Iyer et al., 2020) with lipids resolved in a comparable conformation to that found for RyR1 inactivated. Corresponding residues of interest are indicated. The + sign indicates transmembrane helices of the neighboring subunit.

**Figure 5—video 1.** Gating-induced conformational changes in nanodisc. Conformational change in upper nanodisc belt while morphing from RyR1-ACP/Ca^2+^ inactivated to open conformations. The nanodisc can be seen expanding when transitioning to the open state. Volumes are low pass filtered and helices are displayed at a higher threshold for better visualization.

We resolved two lipid densities at the four inter-subunit interfaces in the RyR1-ACP/Ca^2+^_A_-inactivated CD-TMD-focused map (Figure 5B). The two lipid densities, observed up to 8σ map significance, spanned ~20 Å across the inner leaflet of the SR membrane, at a hydrophobic pocket in the domain-swapped inter-subunit space formed by the S1–S4 bundle and core TMD helices (S5 and S6) (Figure 5B and C). One density (lipid 1), encompassing two fatty acyl tail moieties of 16 and 11 carbons with a polar head compatible with a phosphatidylcholine molecule, was resolved near S5/S6 (Figure 5C). The lipid likely originates from a longer unsaturated PC (16:0–18:1) used in 50-fold molar excess while embedding RyR1 into nanodiscs. Lipid 2, with a resolved 16-carbon fatty acid tail, was sandwiched between lipid 1 and S3/S4. The lipid’s fatty-acyl tails interact extensively with 21 amino acids in the lipophilic pocket formed by S3/S4 and S5/S6 of neighboring subunits, while the density corresponding to the lipid head moiety was positioned within 5 Å from Tyr4629, Asn4857, and Tyr4909 (Figure 5C). Together, the lipids covered ~622 Å^2^ of the 1990 Å^2^ surface area of the S3/S4-S5/S6 subunit interface forming a hydrophobic crevice (Figure 5D). No lipid density was resolved in the RyR1-ACP/Ca^2+^_A_ open map: besides the lower resolution of this map, its crevice is narrower, as S3 and S4 remodeled the lipid-binding pocket by tilting ~4.4° and ~3.9°, respectively. Furthermore, reorientation in Phe4808 (S4) and Tyr4912 (S6) as modeled for the open state would result in steric clash with lipid 2 and lipid 1, respectively (Figure 5—figure supplement 1A and B; lipids superimposed from the RyR1-ACP/Ca^2+^-inactivated reconstruction – shown in Figure 5—figure supplement 1C in the same orientation), rearranging or even excluding the lipids in the open channel. A similar observation was reported for the TRPV3 channel, whereby lipids get squeezed out in going from the closed to the open state (Singh et al., 2018). We did not resolve lipids within the crevice of RyR1-ACP/EGTA, probably owing to the lower resolution of this reconstruction (4.30 Å), but examination of the crevice indicates no expected hindrance to lipid entry offered by residues Phe4808 and Tyr4912 in the closed state (Figure 5—figure supplement 1D). Moreover, we resolved lipids in a higher-resolution (3.27 Å) 3D reconstruction of closed RyR reconstituted in lipids, in this case for the RyR2 isoform (Figure 5—figure supplement 1E; PDB ID: 6WOU; Iyer et al., 2020). The analogous appearance of the lipid densities in the two isoforms is remarkable (three tubular densities with similar lengths and orientations; compare Figure 5—figure supplement 1C and E), suggesting that the interaction of lipids with the RyR TMD crevice is conserved among isoforms, and characteristic of closed and inactivated states.

### Plasticity of transmembrane helices and stability of the luminal mouth of the channel

**Figure 6.**
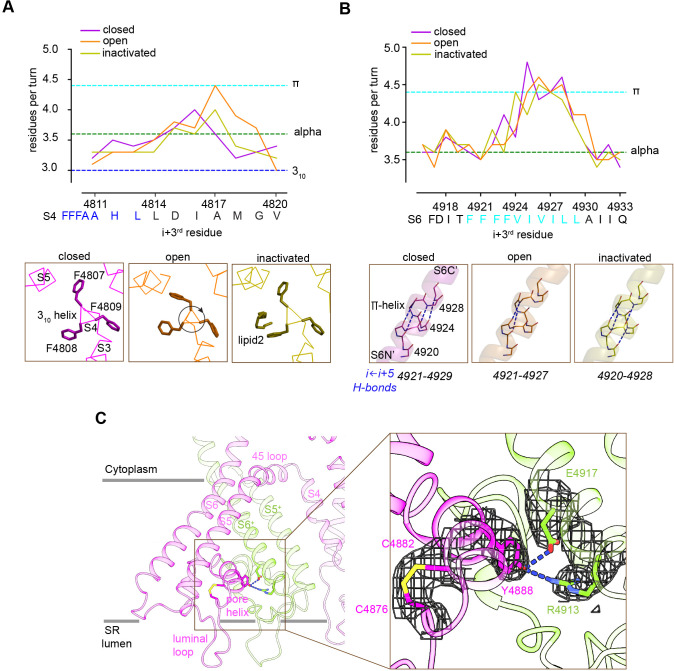
Local conformational changes in the transmembrane helices among closed, open, and inactivated conformations. (**A**) Top: residues per turn for S4 reveals a 3_10_ helix (4807–4813; blue dashed line) at its N′, and helical changes among the three conformations. Residues per turn are computed for a 3-residue moving window. Bottom: a segment of S4 displaying 3_10_ helix in FFF (4807–4809). Uncoiling of the S4-C′ in the open structure rotates the FFF region, including Phe4808, which interacts with lipid 2 in the inactivated structure. (**B**) Top: the N′ region of S6 (residues 4920–4928) contains a tetra Phe motif (4920–4923) followed by a π-helix region. Bottom: the backbone hydrogen-bonding network in the π-helix region in the three distinct conformations; dashed lines show i ← *i + 5* hydrogen bonds. (**C**) A Cys4876-Cys4882 disulfide bond formed between the luminal loop (4860–4878) and the pore helix (4879–4893) of the same subunit, and predicted hydrogen bonds between the P-loop (pore helix) and S6 from the neighboring subunit likely contribute to stabilize the luminal mouth of RyR1. The cryo-EM densities are shown in mesh.

The TMD helices were examined in the membrane-like environment provided by the nanodisc. Significant perturbations in the α-helical configuration were observed. S4 displays near-3_10_ helix in residues 4807–4813 and (only for the open structure) near-π-helix in residues 4814–4819. S5 displays π-helix in residues 4856–4859. S6 displays π-helix in residues 4921–4928 of S6 (Figure 6A and B). In S4, comparison of the three conformations suggests that the degree of over-coiling of the N′ half of S4 is balanced by the uncoiling of its C′ half to near-π-helix in the open structure (Figure 6A), which causes the reorientation of Phe4808 into the hydrophobic crevice. In S6, the π-helix region introduces non-canonical i ← i + 5 hydrogen bonding that is energetically less stable than a regular α-helix (Fodje and Al-Karadaghi, 2002; Kumar and Bansal, 2015; Figure 6B), and may facilitate the flexibility of S6 required for its structural transitions.

The three segments departing from α-helical configuration contain Phe clusters (FFF 4807–4809, FF 4858–4859, FFFF 4920–4923) conserved in all RyR isoforms and IP_3_R intracellular Ca^2+^ release channels; with some of them lining the hydrophobic crevice and forming contacts with the lipids (Figure 5C). Deletion of FF 4923–4924 (human sequence; equivalent to rabbit FF 4922–4923) in S6 of RyR1, which is associated to fetal akinesia deformation syndrome or FADS, resulted in loss of Ca^2+^ conductance (Xu et al., 2020), which further supports a role for the Phe clusters in the stability of the pore.

The SR luminal loop (4860–4878) between S5 and the P-loop helix (also termed pore helix; residues 4879–4893), proposed to act as a luminal Ca^2+^ sensor (Sitsapesan and Williams, 1995) and as an anchorage point for proteins within the SR lumen (Beard et al., 2009), contains six acidic residues (EDEDEPD 4867–4873). This luminal loop is separated from the rest of the TMD, and its acidic residues point to the lumen interior. As our experimental high Ca^2+^ concentrations correspond to these in the SR lumen, this conformation of the luminal loop (Figure 6C) is probably a close representation of the native state. The higher-resolution RyR1-ACP/Ca^2+^-inactivated map revealed a disulfide bond (Cys4876-Cys4882) between S5 and the P-loop (Figure 6C), and two inter-subunit hydrogen bonds (Tyr4888-Arg4913 and Tyr4888-Asp4917) between the P-loop and S6. These interactions may stabilize the luminal mouth of the channel, keeping the proper arrangement of the four SR luminal loops, as well as holding S6 through its conformational transitions.

## Discussion

The goal of this work was to understand the mechanism of Ca^2+^-induced inactivation of RyR1 from a structural point of view. Although maximum inhibition occurred at 10 mM Ca^2+^, we selected a concentration closer to the highest Ca^2+^ concentration in physiological compartments, around 1–2 mM free Ca^2+^ (in the SR lumen, cytoplasmic Ca^2+^ nanodomains, and extracellular medium). At 2 mM free Ca^2+^, we discerned two classes within each of the two datasets, with their ion gate either in an open or a closed conformation on account of direct pore analysis. The concentration of Ca^2+^ used, 2 mM, is near the IC_50_ of this cation determined experimentally in the [^3^H]ryanodine-binding assays in the presence of ACP (the ATP analogue used in our studies), a concentration at which Ca^2+^-activated and Ca^2+^-inactivated RyR1 conformations should coexist. Accordingly, we propose that they correspond with the open-pore and closed-pore conformations observed by cryo-EM, respectively.

Earlier 3D structures of RyR in the open state required synthetic activators such as caffeine (des Georges et al., 2016), which by itself suffices to activate the channel (Xu et al., 2018), or PCB95 (Bai et al., 2016; Samsó et al., 2009). Our cryo-EM maps of RyR1 and lipid were obtained in the absence of any nonphysiological activator, and in a nanodisc environment. Given the lipidic environment provided by the nanodisc and the lack of exogenous activators, the structure of RyR1-ACP/Ca^2+^ open (reproduced in two independent datasets, A and B) obtained in the presence of Ca^2+^ and ACP provides an accurate account of the native open state. Overall, our open-state structure validates the previous RyR1 open structures obtained using exogenous activators.

The RyR1-ATP/caffeine/Ca^2+^ dataset, obtained in the presence of 30 µM Ca^2+^, 2 mM ATP, and 5 mM caffeine, also yielded a class with a closed pore, which was termed ‘primed’ (des Georges et al., 2016). An apparently puzzling result is that our structure of RyR1-ACP/Ca^2+^ inactivated is similar to the primed conformation. Both consist of an occupied Ca^2+^-binding site with a closed pore. The flexion angle of the primed structure (e.g., PDB ID: 5TAQ), around –3°, is also more akin to an open than a closed state. In these studies, caffeine was used as a means to attain the open state of the channel as caffeine sensitizes RyR1 to Ca^2+^ and increases opening probability. However, caffeine also affects inactivation and shifts the entire bell-shaped curve of RyR1 activation as a function of Ca^2+^ concentration to lower Ca^2+^ concentrations: from a maximum at 30 µM Ca^2+^, to well below 10 µM Ca^2+^ in the presence of saturating caffeine (Meissner et al., 1997). Thus, 30 µM Ca^2+^ in the presence of caffeine is on the descending branch of the curve, which corresponds to partially inactivating conditions. According to this, the RyR1 channel conformation qualified as ‘primed’ in the earlier study is, very likely, inactivated and appears to correspond to the RyR1-ACP/Ca^2+^-inactivated conformation characterized here.

Activation and inactivation appear to proceed under an integrated mechanism, where inactivation depends on prior activation. Both RyR1 open and inactivated conformations showed that Ca^2+^ bound at the high-affinity site in the CD/CTD interface, which joined these and their proximal domains (EF, U-motif, and S6C′) into a more rigid central block. In activation, CD and CTD rotate toward each other closing around the Ca^2+^ ion; the rotation of the CTD pulls S6C′ outwards, opening the pore. In inactivation, the central block rotates further into the arc initiated by the CD alone, which now pushes the S6 helices toward the central axis. The movement is similar to pushing down the levers of a winged corkscrew while pushing toward the central rod (see schematics in Figure 2D), closing the channel in a distinctive conformation different from the EGTA-closed resting state. This state, with a closed pore and Ca^2+^ bound to the activation site, can no longer be activated by Ca^2+^, which is the hallmark of an inactivated state.

Comparison of the open, closed, and inactivated conformations uncovers other novel features. A strong density connected to ACP under the high Ca^2+^ conditions, which we attribute to hydrated Ca^2+^, progressively supports more interactions with the CTD and S6, increasing cohesiveness of the central block (in the order inactivated >open >closed; Figure 4), which could help to explain the more robust Ca^2+^- induced inactivation reported in the presence of ATP (Sitsapesan and Williams, 2000). In this context, it is important to keep in mind that Mg^2+^ at physiological concentration also binds to the nucleotide-binding pocket of RyR1, which we can confirm with our 3D reconstruction of RyR1-ACP/Mg^2+^ (PDB ID: 7K0S). While in general ATP has a higher affinity for Mg^2+^ than for Ca^2+^, the Ca^2+^ occupancy of this site in the inactivated state is significant (RMSD 15; Figure 4—figure supplement 1). Thus, it is difficult to predict how both cations may compete for the same site, something that will require further study. Besides the high-affinity Ca^2+^-binding site and the nucleotide-binding site, there were no other obvious densities in the resolved regions of the RyR1 structure that could account for a bound Ca^2+^ ion, or clusters of negatively charged residues that could support further Ca^2+^-mediated conformational changes.

Mg^2+^ is a potent inhibitor of RyR1, and a mechanism of Mg^2+^ competition for the Ca^2+^ binding site was proposed (Lamb and Stephenson, 1991; Meissner et al., 1986). We carried out a 3D reconstruction of RyR1-ACP/Mg^2+^ (PDB ID: 7K0S) and found that, at a physiological concentration of Mg^2+^, the high-affinity Ca^2+^-binding site remains empty. Importantly, the Mg^2+^-inhibited conformation of RyR1 is clearly distinct from the Ca^2+^-inactivated RyR1 and bears more resemblance to the EGTA-closed conformation.

The EF hand domain did not have Ca^2+^ bound in the high Ca^2+^ datasets in keeping with the low affinity of Ca^2+^ to this site (Xiong et al., 1998). However, this protruding domain, with its sequence between the CD and the U-motif, appears to play a distinctive role in inactivation that derives from its further anticlockwise and out-of-plane rotations with respect to the open state (Figure 3). This conformation brings the EF hand domain in contact with the cytoplasmic loop between the S2 and S3 transmembrane helices of the neighboring subunit, forming two inter-subunit electrostatic interactions (Figure 3). One possible scenario is that the energy landscape of the open channel allows for overshoot of the opening motion, allowing interaction of the EF hand and S2–S3 loop domains by such salt bridges. These appear to stabilize the inactivated conformation by providing an extra linkage between subunits, and between the cytoplasmic assembly and TMD. Functional studies of MH mutations in the S2–S3 loop and EF hand domains identified a subset of mutations that altered RyR1 function by impairing Ca^2+^ inactivation (Gomez et al., 2016). Interestingly, only this subset of residues, when mutated, would impair the inter-domain interactions that we identified, which supports the proposed role of the two salt bridges (Glu4075-Arg4736 and Lys4101-Asp4730) in stabilizing the inactivated state of RyR1.

In the TMD, owing to the nanodisc environment, two lipids in a crevice between S3/S4 of one subunit and S5/S6 of the neighboring subunit were resolved in the inactivated state. The hydrophobic nature of this crevice suggests that lipids may help to stabilize the TMD in the inactivated state. Although we could not resolve lipids in the closed state probably owing to the lower resolution of this 3D reconstruction, we observed lipids for the RyR2 isoform reconstituted in nanodiscs under closed-state conditions (Iyer et al., 2020). The orientation of the two lipids in the two isoforms of RyR is identical. In the open state, the hydrophobic crevice is narrower, and Phe4808 of S4 adopts a different orientation that would clash with lipid 2, suggesting rearrangement in the open state. A similar lipid exclusion of bound lipid in the open channel was reported for the TRPV3 channel (Singh et al., 2018). Thus, the closed-state-dependent occupation of the TMD crevice by lipids could constitute a common feature among 6-TMD Ca^2+^ channels.

Departure from α-helix geometry was also present in segments of S4 (near-3_10_ helix and near-π-helix), and S5 and S6 (π-helix). In the case of S4, helical structural transitions correlate with the closed, open, and inactivated conformations. The dynamism of the transmembrane helices through the gating transitions appears to be supported by anchoring of Phe motifs in S4, S5, and S6 to lipids in the membrane. In addition, inter-subunit interactions including disulfide bridges inter-connect the 4860–4878 SR luminal loop, the P-loop, and S6N′ around the luminal mouth of the channel where the four protomers converge.

Functional studies employing single channels embedded in lipid bilayers show rapid channel inactivation following an RyR1 Ca^2+^ release event. Therefore, after RyR1 opening, a refractory period is needed to relieve inactivation and recover the ability to activate again (Laver and Lamb, 1998; Ríos et al., 2008; Schiefer et al., 1995; Sitsapesan and Williams, 2000). The 3D reconstructions reported here provide a structural basis for this refractoriness: we hypothesize that the 3D reconstructions reported here, combined with the time course of Ca^2+^ release, provide a mechanism for this refractoriness as follows. When RyR1 opens, Ca^2+^ concentration in its surrounding nanodomain increases rapidly, and time from Ca^2+^ release onset also increases. Both augment occupancy of the high-affinity Ca^2+^-binding site and the probability of a full conformational change of the CD/CTD block induced by Ca^2+^, which in turn increases the successful formation of the inter-subunit salt bridges, ‘sealing’ the transition of the channel to the inactivated state. At this time, the Ca^2+^-inactivated state is in a distinctive locked closed conformation while the high-affinity Ca^2+^-binding site is still occupied. This renders this closed conformation unable to be activated by Ca^2+^ as long as Ca^2+^ occupies the high-affinity site. In this way, Ca^2+^ permeation through the RyR1 provides negative feedback through the same binding site. Ca^2+^-dependent inactivation is often observed in Ca^2+^ permeation pathways, probably to limit cytosolic Ca^2+^ overload that could be detrimental and life-threatening (Dick et al., 2016; Gomez et al., 2016). Together with previous investigations (Gomez et al., 2016; Gomez and Yamaguchi, 2014), we propose a structural mechanism for how naturally occurring mutations disturb RyR1 inactivation producing Ca^2+^ dysregulation and muscle disease. Overall, our study provides a structural basis to understand the transitions from the closed, to the open, and then to the Ca^2+^-inactivated state of the RyR1 at high resolution.

## Materials and methods

**Key resources table:** 

Reagent type (species) or resource	Designation	Source or reference	Identifiers	Additional information
Strain, strain background (*Oryctolagus cuniculus,* mixed gender)	New Zealand White	Charles River	NZW 052	
Recombinant DNA reagent	Membrane scaffold protein plasmid pMSP1E3D1	Addgene	Cat# 20066	
Chemical compound, drug	Ryanodine, [9,21-^3^H(N)]-, 250 µCi	PerkinElmer	Part# NET950250UC	
Chemical compound, drug	Adenosine-5'-[(α,β)-methyleno]diphosphate, Sodium salt	Jena Bioscience	Cat# NU-420-25	
Chemical compound, drug	16:0-18:1 PC(POPC)	Avanti Polar Lipids	Cat# 850457	
Chemical compound, drug	3-[(3-Cholamidopropyl) dimethylammonio]–1-propanesulfonate (CHAPS)	Sigma-Aldrich	Cat# 220201	
Chemical compound, drug	HiTrap Heparin HP	Cytiva	Cat# 17-0406-01	
Chemical compound, drug	L-α-phosphatidylcholine	Sigma-Aldrich	Cat# P3644	
Chemical compound, drug	Sucrose	Sigma-Aldrich	Cat# S9378	
Software, algorithm	Maxchelator	Bers et al., 2010	https://somapp.ucdmc.ucdavis.edu/pharmacology/bers/maxchelator/CaMgATPEGTA-TS.htm	
Software, algorithm	MotionCor2	Zheng et al., 2017		
Software, algorithm	Gctf	Zhang, 2016		
Software, algorithm	RELION-3.0	Scheres, 2012		
Software, algorithm	PHENIX	Afonine et al., 2018	http://www.phenix-online.org/	
Software, algorithm	HELANAL	Bansal et al., 2000	http://nucleix.mbu.iisc.ernet.in/helanalplus/index.html	
Software, algorithm	Coot	Emsley et al., 2010	https://www2.mrc-lmb.cam.ac.uk/personal/pemsley/coot/	
Software, algorithm	UCSF Chimera	Pettersen et al., 2004	https://www.cgl.ucsf.edu/chimera/	
Software, algorithm	UCSF ChimeraX	Pettersen et al., 2021	https://www.cgl.ucsf.edu/chimerax/	
Software, algorithm	PyMOL	Schrodinger, 2015	https://pymol.org/2/	
Software, algorithm	HOLE	Smart et al., 1993	http://www.holeprogram.org/	
Software, algorithm	Adobe Creative Cloud	Adobe	https://www.adobe.com/creativecloud/	
Other	UltraAufoil –1.2/1.3 Holey-Gold 300 mesh grids	Quantifoil, Germany	https://www.quantifoil.com/products/ultrafoil	

### Reagents

All chemicals were purchased from Thermo Fisher or Sigma-Aldrich except where indicated.

### [^3^H]Ryanodine binding

RyR1 activity was estimated by measuring the extent of bound ^3^[H]ryanodine in microsomes isolated from rabbit skeletal muscle when incubated with free Ca^2+^ alone (10 μM to 2 mM range), or in the presence of 2 mM ATP or 2 mM AMP-PCP (ACP) sodium salts. Concentrations of total Ca^2+^ added to the reaction mixture were estimated in Maxchelator (https://somapp.ucdmc.ucdavis.edu/pharmacology/bers/maxchelator). Preincubated membrane vesicles (~40 μg) were allowed to bind 5 nM [^3^H]ryanodine (PerkinElmer) in a buffer containing 50 mM MOPS (pH 7.4), 0.15 M KCl, 0.3 mM EGTA, protease inhibitors, and 2 mM DTT for 3 hr at 37°C. Sample aliquots were diluted sevenfold with an ice-cold wash buffer (0.1 M KCl) before placing onto Whatman GF/B filter papers in a vacuum-operated filtration apparatus. The remaining radioactivity in the filter papers after washing three times with the wash buffer was measured by liquid scintillation counting. Nonspecific ryanodine binding was estimated in the presence of 250 μM unlabeled ryanodine (Calbiochem) and subtracted from the total binding. Data represent the mean specific [^3^H]ryanodine binding from four independent experiments.

### Purification of RyR1 from rabbit skeletal muscle and reconstitution into nanodiscs

Microsomes were purified from rabbit back and hind leg muscles through differential centrifugation as previously described (Hu et al., 2021; Samsó et al., 2009). 100 mg frozen membranes were thawed and solubilized in buffer A containing 20 mM MOPS pH 7.4, 1 M NaCl, 9.2% (w/v) CHAPS, 2.3% (w/v) phosphatdylcholine (PC; Sigma), 2 mM DTT, and protease inhibitor cocktail for 15 min at 4°C. The solubilized membranes were centrifuged at 100,000 × *g* for 60 min and the pellet was discarded. Supernatant was layered onto 10–20% (w/v) discontinuous sucrose gradients, prepared in buffer B (buffer A plus 0.5% CHAPS and 0.125% PC). The layered sucrose gradient tubes were ultracentrifuged at 120,000 × *g* for ~20 hr at 4°C to allow RyR1 separation. Fractions containing >95% pure RyR1 were pooled and further purified with a HiTrap Heparin HP Agarose column (GE Healthcare) after a fivefold dilution in salt-free buffer and filtration steps. RyR1 was eluted with buffer B containing 0.9 M NaCl, after washing with 20 column volumes of buffer B with 200 mM NaCl. Peak fractions were flash-frozen and stored at –80°C until reconstitution into nanodiscs and cryo-EM. 1.5–2 mg of RyR1 was purified from 100 mg of SR membrane vesicles. RyR1 purity was estimated with 12.5% SDS-PAGE and negative staining with 0.75% uranyl formate. Protein concentration in purified microsomes and RyR1 fractions was measured with Quick Start Bradford Protein Assay (Bio-Rad). The plasmid encoding for MSP1E3D1, pMSP1E3D1, was purchased from Addgene, and recombinant MSP1E3D1 was purified in *Escherichia coli* using the manufacturer’s instructions. RyR1-nanodiscs were obtained by mixing purified RyR1, MSP1E3D1, and POPC (Avanti polar lipids) at a 1:2:50 molar ratio. The mixture was incubated for 1 hr 30 min at 4°C before an overnight dialysis in a CHAPS-free buffer (20 mM MOPS pH 7.4, 635 mM KCl, 2 mM DTT), which contained either 1 mM EGTA +1 mM EDTA for the control ‘RyR1-ACP/EGTA’ dataset or 3.7 mM CaCl_2_ for the RyR1-ACP/Ca^2+^ dataset. The dialyzed RyR1-nanodisc preparations were incubated with ACP (sodium salt) for 30 min prior to plunge freezing, at concentrations of 5 mM ACP (control RyR1-ACP/EGTA dataset) or 2 mM ACP (RyR1-ACP/Ca^2+^ datasets). Free Ca^2+^ was estimated with Maxchelator. Integrity of the nanodisc-embedded channels was examined by negative staining.

### Cryo-EM grid preparation and data acquisition

Cryo-EM grids were cleaned with a customized protocol (Passmore and Russo, 2016) prior to glow discharge. Aliquots of 1.25–1.5 µl RyR1-nanodisc were applied onto each side of glow-discharged 300 mesh UltraAufoil –1.2/1.3 Holey-Gold (Quantifoil, Germany). The grids were blotted for 1–1.5 s with an ashless Whatman Grade 540 filter paper in a Vitrobot Mark IV (Thermo Fisher Scientific) and rapidly plunged into liquid ethane. Grid quality and RyR1 sample distribution were assessed on a Tecnai F20 (Thermo Fisher Scientific) electron microscope. Data acquisition was carried out in a Titan Krios transmission electron microscope (Thermo Fisher Scientific) operated at 300 kV and counting mode, with a K3 or K2 detector (Gatan) for the ACP/Ca^2+^_A_ and ACP/Ca^2+^_B_ datasets, respectively. A Gatan Quantum Energy Filter (GIF) with a slit width of 20 eV was employed. The ACP/EGTA dataset was collected on a K2 detector and a 20 eV GIF. Datasets were collected in automated mode with the program Latitude (Gatan) with a cumulative electron dose of 70 e^-^/Å^2^ applied over 50–60 frames. Image acquisition parameters for RyR1-ACP/EGTA, RyR1-ACP/Ca^2+^_A_, and RyR2-ACP/Ca^2+^_B_ datasets are summarized in Table 1.

### Single-particle image processing

Gain reference normalization, movie frame alignment, dose weighting, and motion correction of the collected movie stacks were carried out with Motioncor2 (Zheng et al., 2017). Contrast transfer function parameters were estimated from non-dose-weighted motion-corrected images using Gctf (Zhang, 2016). All subsequent image processing operations were carried out using dose-weighted, motion-corrected micrographs in RELION 3.0 (Scheres, 2012). The micrographs were low-pass filtered to 20 Å before automated particle picking. 2D class average templates for autopicking were generated by reference-free 2D classification of 1000 manually picked particles. Autopicked particles with ethane and hexagonal ice-contaminated areas were removed by visual inspection. Particle image sub-stacks required for the focused reconstructions residues 3668–5037, encompassing the CD, U-motif, TMD, and CTDs, were generated using a signal subtraction procedure employed in relion_project module (Bai et al., 2015). Particle image stacks of quarter sub-volumes of RyR1 corresponding to a single subunit were generated by particle subtraction following a symmetry expansion step with relion_particle_symmetry_expand tool (Bai et al., 2015; Scheres, 2016). Composite tetrameric maps were generated from the symmetry-expanded monomeric maps with Chimera (Pettersen et al., 2004) vop maximum tool. B-factor applied to the reconstructed maps was estimated with relion_postprocess. Analysis of the TMD was carried out on a focused map of the CD-TMD region. Unfiltered half maps obtained from the final 3D-refinement step in RELION 3.0 were further density modified with PHENIX.Resolve (Terwilliger et al., 2020). The reported resolutions of the cryo-EM maps are based on FSC 0.143 criterion (Scheres and Chen, 2012). Local resolution was estimated with ResMap (Kucukelbir et al., 2014). Pixel size calibration of postprocessed maps was carried out using real space correlation metric of UCSF Chimera based on a published RyR1 cryo-EM map (des Georges et al., 2016). Pixel size maxima of 1.07, 1.105, and 1.07 Å were obtained in RyR1-ACP/EGTA, RyR1-ACP/Ca^2+^_A_, and RyR1-ACP/Ca^2+^_B_ respectively. Image processing schemes of the RyR1-ACP/Ca^2+^_A_, RyR1-ACP/Ca^2+^_B,_ and RyR1-ACP/EGTA datasets are summarized in Figure 1—figure supplement 1, Figure 1—figure supplement 3, and Figure 1—figure supplement 4, respectively.

### Model building and structure refinement

The cryo-EM-based atomic models of RyR1 (PDB ID: 5tb3 for RyR1-ACP/EGTA, RyR1-ACP/Ca^2+^ inactivated and 5ta3 for RyR1-ACP/Ca^2+^ open) were used as the initial models for model building. The best resolved symmetry-expanded cryo-EM map for a single subunit in inactivated or open conformation was docked within a RyR1 monomer model with Chimera Fit in map tool. Local density fit of the RyR1 sequence was improved over an iterative process of amino acid fitting in Coot (Emsley et al., 2010) alternated with real space refinement in PHENIX (Afonine et al., 2018). Four copies of the monomers were docked to the whole RyR1 reconstructions. Real space refinement of the tetrameric models was carried out with secondary structure and Ramachandran restraints. Further manual fitting of the CD, TMD, and CTD (3668–5037) of RyR1 was carried out in Coot. Comprehensive model validation was carried out with PHENIX and PDB validation server at https://validate-rcsb-2.wwpdb.org/ and is summarized in Table 2. Molecular lipophilicity potential surfaces were drawn in ChimeraX (Ghose et al., 1998; Laguerre et al., 1997; Pettersen et al., 2021). Figures were generated with PyMOL (Schrodinger, 2015) and Chimera programs (Pettersen et al., 2004; Pettersen et al., 2021).

### Pore radius and helical geometry measurements

Pore radii were measured for the refined atomic model coordinates of the RyR1 pore region (residues 4821–5037) with the HOLE program (Smart et al., 1993). Dot surfaces representing the channel ion permeation pathway were generated with HOLE implemented in Coot, which were reformatted to enable visualization in UCSF Chimera. Residues per turn of S4 and S6 transmembrane helices of RyR1 were calculated with HELANAL (Bansal et al., 2000).

### Flexion angle measurement

The cytoplasmic shell flexion angles of RyR1 in different conformations were estimated using the procedure indicated in Steele and Samsó, 2019 with minor changes. The angle was calculated between a diagonal running from the N-terminal domain (residue 348) to the P1 domain (residue 984) from the same subunit, and the horizontal plane.

### Data availability

Tetrameric and focused cryo-EM maps of RyR1-ACP/EGTA, RyR1-ACP/Ca^2+^ inactivated, and RyR1-ACP/Ca^2+^ open have been deposited in the Electron Microscopy Databank (EMDB) with the following accession codes: 22616, 22597 (RyR1-ACP/EGTA tetrameric and focused maps), 25828 (RyR1-ACP/Ca^2+^_A_ inactivated; focused map as an additional map), 25830, 25831, 25832 (three subclasses of RyR1-ACP/Ca^2+^_A_ inactivated), 25829 (RyR1-ACP/Ca^2+^_A_ open), and 25833 (RyR1-ACP/Ca^2+^_B_ inactivated). Atomic models generated from the cryo-EM maps have been deposited in the RCSB PDB database with the following accession codes: 7K0T (RyR1-ACP/EGTA), 7TDG (RyR1-ACP/Ca^2+^_A_ inactivated), 7TDJ, 7TDI, 7TDK (three subclasses of RyR1-ACP/Ca^2+^_A_ inactivated), and 7TDH (RyR1-ACP/Ca^2+^_A_ open).

## Data Availability

The cryo-EM maps and models are available in the EMDB and PDB databases. The following datasets were generated: NayakAR SamsóM 2021Cryo-EM structure of rabbit RyR1 in the presence of AMP-PCP in nanodiscRCSB Protein Data BankPDB-7K0T NayakAR SamsóM 2021Cryo-EM structure of rabbit RyR1 in the presence of AMP-PCP in nanodiscElectron Microscopy Data BankEMD-22616 NayakAR SamsóM 2021Focused cryo-EM map of rabbit RyR1 central and transmembrane domains in the presence of AMP-PCP in nanodiscElectron Microscopy Data BankEMD-22597 NayakAR SamsóM 2022Rabbit RyR1 with AMP-PCP and high Ca2+ embedded in nanodisc in inactivated conformationRCSB Protein Data BankPDB-7TDG NayakAR SamsóM 2022Rabbit RyR1 with AMP-PCP and high Ca2+ embedded in nanodisc in inactivated conformation, (Dataset-A)Electron Microscopy Data BankEMD-25828 NayakAR SamsóM 2022Rabbit RyR1 with AMP-PCP and high Ca2+ embedded in nanodisc in closed-inactivated conformation class 1 (Dataset-A)RCSB Protein Data BankPDB-7TDJ NayakAR SamsóM 2022Rabbit RyR1 with AMP-PCP and high Ca2+ embedded in nanodisc in closed-inactivated conformation class 1 (Dataset-A)Electron Microscopy Data BankEMD-25831 NayakAR SamsóM 2022Rabbit RyR1 with AMP-PCP and high Ca2+ embedded in nanodisc in closed-inactivated conformation class 2 (Dataset-A)RCSB Protein Data BankPDB-7TDI NayakAR SamsóM 2022Rabbit RyR1 with AMP-PCP and high Ca2+ embedded in nanodisc in closed-inactivated conformation class 2 (Dataset-A)Electron Microscopy Data BankEMD-25830 NayakAR SamsóM 2022Rabbit RyR1 with AMP-PCP and high Ca2+ embedded in nanodisc in closed-inactivated conformation class 3 (Dataset-A)RCSB Protein Data BankPDB-7TDK NayakAR SamsóM 2022Rabbit RyR1 with AMP-PCP and high Ca2+ embedded in nanodisc in closed-inactivated conformation class 3 (Dataset-A)Electron Microscopy Data BankEMD-25832 NayakAR SamsóM 2022Rabbit RyR1 with AMP-PCP and high Ca2+ embedded in nanodisc in open conformationRCSB Protein Data BankPDB-7TDH NayakAR SamsóM 2022Rabbit RyR1 with AMP-PCP and high Ca2+ embedded in nanodisc in open conformationElectron Microscopy Data BankEMD-25829 NayakAR SamsóM 2022Rabbit RyR1 with AMP-PCP and high Ca2+ embedded in nanodisc in inactivated conformation (Dataset-B)Electron Microscopy Data BankEMD-25833

## Additional files

Transparent reporting form

## References

[bib1] Afonine PV, Klaholz BP, Moriarty NW, Poon BK, Sobolev OV, Terwilliger TC, Adams PD, Urzhumtsev A (2018). New tools for the analysis and validation of cryo-EM maps and atomic models. Acta Crystallographica. Section D, Structural Biology.

[bib2] Albrecht MA, Colegrove SL, Hongpaisan J, Pivovarova NB, Andrews SB, Friel DD (2001). Multiple modes of calcium-induced calcium release in sympathetic neurons I: attenuation of endoplasmic reticulum Ca2+ accumulation at low [Ca2+](i) during weak depolarization. The Journal of General Physiology.

[bib3] Alkhunaizi E, Shuster S, Shannon P, Siu VM, Darilek S, Mohila CA, Boissel S, Ellezam B, Fallet-Bianco C, Laberge A-M, Zandberg J, Injeyan M, Hazrati L-N, Hamdan F, Chitayat D (2019). Homozygous/compound heterozygote RYR1 gene variants: expanding the clinical spectrum. American Journal of Medical Genetics. Part A.

[bib4] Arias-Cavieres A, Barrientos GC, Sánchez G, Elgueta C, Muñoz P, Hidalgo C (2018). Ryanodine Receptor-Mediated Calcium Release Has a Key Role in Hippocampal LTD Induction. Frontiers in Cellular Neuroscience.

[bib5] Bagur R, Hajnóczky G (2017). Intracellular Ca(2+) Sensing: Its Role in Calcium Homeostasis and Signaling. Molecular Cell.

[bib6] Bai X, Rajendra E, Yang G, Shi Y, Scheres SHW (2015). Sampling the conformational space of the catalytic subunit of human γ-secretase. eLife.

[bib7] Bai XC, Yan Z, Wu J, Li Z, Yan N (2016). The Central domain of RyR1 is the transducer for long-range allosteric gating of channel opening. Cell Research.

[bib8] Bansal M, Kumar S, Velavan R (2000). HELANAL: A Program to Characterize Helix Geometry in Proteins. Journal of Biomolecular Structure & Dynamics.

[bib9] Beard NA, Wei L, Dulhunty AF (2009). Control of muscle ryanodine receptor calcium release channels by proteins in the sarcoplasmic reticulum lumen. Clinical and Experimental Pharmacology & Physiology.

[bib10] Bers DM (2002). Cardiac excitation–contraction coupling. Nature.

[bib11] Bers DM, Patton CW, Nuccitelli R (2010). A Practical Guide to the Preparation of Ca2+ Buffers. Methods in Cell Biology.

[bib12] Bezprozvanny I, Watras J, Ehrlich BE (1991). Bell-shaped calcium-response curves of lns(l,4,5)P3- and calcium-gated channels from endoplasmic reticulum of cerebellum. Nature.

[bib13] Bouchard R, Pattarini R, Geiger JD (2003). Presence and functional significance of presynaptic ryanodine receptors. Progress in Neurobiology.

[bib14] Chen SR, Li X, Ebisawa K, Zhang L (1997). Functional characterization of the recombinant type 3 Ca2+ release channel (ryanodine receptor) expressed in HEK293 cells. The Journal of Biological Chemistry.

[bib15] Chen W, Kudryashev M (2020). Structure of RyR1 in native membranes. EMBO Reports.

[bib16] Chirasani VR, Xu L, Addis HG, Pasek DA, Dokholyan NV, Meissner G, Yamaguchi N (2019). A central core disease mutation in the Ca(2+)-binding site of skeletal muscle ryanodine receptor impairs single-channel regulation. American Journal of Physiology. Cell Physiology.

[bib17] des Georges A, Clarke OB, Zalk R, Yuan Q, Condon KJ, Grassucci RA, Hendrickson WA, Marks AR, Frank J (2016). Structural Basis for Gating and Activation of RyR1. Cell.

[bib18] Dick IE, Joshi-Mukherjee R, Yang W, Yue DT (2016). Arrhythmogenesis in Timothy Syndrome is associated with defects in Ca(2+)-dependent inactivation. Nature Communications.

[bib19] Du GG, Imredy JP, MacLennan DH (1998). Characterization of recombinant rabbit cardiac and skeletal muscle Ca2+ release channels (ryanodine receptors) with a novel [3H]ryanodine binding assay. The Journal of Biological Chemistry.

[bib20] Emsley P, Lohkamp B, Scott WG, Cowtan K (2010). Features and development of Coot. Acta Crystallographica. Section D, Biological Crystallography.

[bib21] Flucher BE, Franzini-Armstrong C (1996). Formation of junctions involved in excitation-contraction coupling in skeletal and cardiac muscle. PNAS.

[bib22] Fodje MN, Al-Karadaghi S (2002). Occurrence, conformational features and amino acid propensities for the π-helix. Protein Engineering.

[bib23] Ghose AK, Viswanadhan VN, Wendoloski JJ (1998). Prediction of Hydrophobic (Lipophilic) Properties of Small Organic Molecules Using Fragmental Methods: An Analysis of ALOGP and CLOGP Methods. The Journal of Physical Chemistry A.

[bib24] Gomez AC, Yamaguchi N (2014). Two regions of the ryanodine receptor calcium channel are involved in Ca(2+)-dependent inactivation. Biochemistry.

[bib25] Gomez AC, Holford TW, Yamaguchi N (2016). Malignant hyperthermia-associated mutations in the S2-S3 cytoplasmic loop of type 1 ryanodine receptor calcium channel impair calcium-dependent inactivation. American Journal of Physiology. Cell Physiology.

[bib26] Hu Y, Iyer KA, Nayak AR, Kurebayashi N, Murayama T, Samsó M (2021). Purification of Recombinant Wild Type and Mutant Ryanodine Receptor Expressed in HEK293 Cell Culture. Bio-Protocol.

[bib27] Iyer KA, Hu Y, Nayak AR, Kurebayashi N, Murayama T, Samsó M (2020). Structural mechanism of two gain-of-function cardiac and skeletal RyR mutations at an equivalent site by cryo-EM. Science Advances.

[bib28] Kucukelbir A, Sigworth FJ, Tagare HD (2014). Quantifying the local resolution of cryo-EM density maps. Nature Methods.

[bib29] Kumar P, Bansal M (2015). Dissecting π-helices: sequence, structure and function. The FEBS Journal.

[bib30] Kushmerick MJ, Moerland TS, Wiseman RW (1992). Mammalian skeletal muscle fibers distinguished by contents of phosphocreatine, ATP, and Pi. PNAS.

[bib31] Laguerre M, Saux M, Dubost JP, Carpy A (1997). MLPP: A Program for the Calculation of Molecular Lipophilicity Potential in Proteins. Pharmacy and Pharmacology Communications.

[bib32] Lamb GD, Stephenson DG (1991). Effects of Mg^2+^ on the control of Ca^2+^ release in skeletal muscle fibers of the toad. The Journal of Physiology.

[bib33] Langer GA, Peskoff A (1996). Calcium concentration and movement in the diadic cleft space of the cardiac ventricular cell. Biophysical Journal.

[bib34] Laskowski RA, Swindells MB (2011). LigPlot+: multiple ligand-protein interaction diagrams for drug discovery. Journal of Chemical Information and Modeling.

[bib35] Laver DR, Roden LD, Ahern GP, Eager KR, Junankar PR, Dulhunty AF (1995). Cytoplasmic Ca2+ inhibits the ryanodine receptor from cardiac muscle. The Journal of Membrane Biology.

[bib36] Laver DR, Lamb GD (1998). Inactivation of Ca2+ release channels (ryanodine receptors RyR1 and RyR2) with rapid steps in [Ca2+] and voltage. Biophysical Journal.

[bib37] Laver DR (2018). Regulation of the RyR channel gating by Ca(2+) and Mg(2). Biophysical Reviews.

[bib38] McCarthy TV, Quane KA, Lynch PJ (2000). Ryanodine receptor mutations in malignant hyperthermia and central core disease. Human Mutation.

[bib39] Meissner G, Darling E, Eveleth J (1986). Kinetics of rapid Ca2+ release by sarcoplasmic reticulum Effects of Ca2+, Mg2+, and adenine nucleotides. Biochemistry.

[bib40] Meissner G, Rios E, Tripathy A, Pasek DA (1997). Regulation of skeletal muscle Ca2+ release channel (ryanodine receptor) by Ca2+ and monovalent cations and anions. The Journal of Biological Chemistry.

[bib41] Meissner G (2017). The structural basis of ryanodine receptor ion channel function. The Journal of General Physiology.

[bib42] Passmore LA, Russo CJ (2016). Specimen Preparation for High-Resolution Cryo-EM. Methods in Enzymology.

[bib43] Pettersen EF, Goddard TD, Huang CC, Couch GS, Greenblatt DM, Meng EC, Ferrin TE (2004). UCSF Chimera--a visualization system for exploratory research and analysis. Journal of Computational Chemistry.

[bib44] Pettersen EF, Goddard TD, Huang CC, Meng EC, Couch GS, Croll TI, Morris JH, Ferrin TE (2021). UCSF ChimeraX: Structure visualization for researchers, educators, and developers. Protein Science.

[bib45] Priori SG, Napolitano C, Tiso N, Memmi M, Vignati G, Bloise R, Sorrentino V, Danieli GA (2001). Mutations in the cardiac ryanodine receptor gene (hRyR2) underlie catecholaminergic polymorphic ventricular tachycardia. Circulation.

[bib46] Renken C, Hsieh C, Marko M, Rath B, Leith A, Wagenknecht T, Frank J, Mannella CA (2009). Structure of frozen-hydrated triad junctions: a case study in motif searching inside tomograms. Journal of Structural Biology.

[bib47] Ríos E, Zhou J, Brum G, Launikonis BS, Stern MD (2008). Calcium-dependent inactivation terminates calcium release in skeletal muscle of amphibians. The Journal of General Physiology.

[bib48] Ríos E (2018). Calcium-induced release of calcium in muscle: 50 years of work and the emerging consensus. The Journal of General Physiology.

[bib49] Samsó M, Feng W, Pessah IN, Allen PD (2009). Coordinated Movement of Cytoplasmic and Transmembrane Domains of RyR1 upon Gating. PLOS Biology.

[bib50] Scheres SHW (2012). RELION: implementation of a Bayesian approach to cryo-EM structure determination. Journal of Structural Biology.

[bib51] Scheres SHW, Chen S (2012). Prevention of overfitting in cryo-EM structure determination. Nature Methods.

[bib52] Scheres SHW (2016). Processing of Structurally Heterogeneous Cryo-EM Data in RELION. Methods in Enzymology.

[bib53] Schiefer A, Meissner G, Isenberg G (1995). Ca2+ activation and Ca2+ inactivation of canine reconstituted cardiac sarcoplasmic reticulum Ca(2+)-release channels. The Journal of Physiology.

[bib54] Schrodinger LLC (2015). PyMOL.

[bib55] Singh AK, McGoldrick LL, Sobolevsky AI (2018). Structure and gating mechanism of the transient receptor potential channel TRPV3. Nature Structural & Molecular Biology.

[bib56] Sitsapesan R, Williams AJ (1995). The gating of the sheep skeletal sarcoplasmic reticulum Ca(2+)-release channel is regulated by luminal Ca2+. The Journal of Membrane Biology.

[bib57] Sitsapesan R, Williams AJ (2000). Do Inactivation Mechanisms Rather than Adaptation Hold the Key to Understanding Ryanodine Receptor Channel Gating. The Journal of General Physiology.

[bib58] Smart OS, Goodfellow JM, Wallace BA (1993). The pore dimensions of gramicidin A. Biophysical Journal.

[bib59] Steele TWE, Samsó M (2019). The FKBP12 subunit modifies the long-range allosterism of the ryanodine receptor. Journal of Structural Biology.

[bib60] Terwilliger TC, Ludtke SJ, Read RJ, Adams PD, Afonine PV (2020). Improvement of cryo-EM maps by density modification. Nature Methods.

[bib61] Tiso N, Stephan DA, Nava A, Bagattin A, Devaney JM, Stanchi F, Larderet G, Brahmbhatt B, Brown K, Bauce B, Muriago M, Basso C, Thiene G, Danieli GA, Rampazzo A (2001). Identification of mutations in the cardiac ryanodine receptor gene in families affected with arrhythmogenic right ventricular cardiomyopathy type 2 (ARVD2). Human Molecular Genetics.

[bib62] Treves S, Jungbluth H, Muntoni F, Zorzato F (2008). Congenital muscle disorders with cores: the ryanodine receptor calcium channel paradigm. Current Opinion in Pharmacology.

[bib63] Tu MK, Levin JB, Hamilton AM, Borodinsky LN (2016). Calcium signaling in skeletal muscle development, maintenance and regeneration. Cell Calcium.

[bib64] Xiong H, Feng X, Gao L, Xu L, Pasek DA, Seok JH, Meissner G (1998). Identification of a Two EF-Hand Ca2+ Binding Domain in Lobster Skeletal Muscle Ryanodine Receptor/Ca2+ Release Channel. Biochemistry.

[bib65] Xu L, Mann G, Meissner G (1996). Regulation of cardiac Ca2+ release channel (ryanodine receptor) by Ca2+, H+, Mg2+, and adenine nucleotides under normal and simulated ischemic conditions. Circulation Research.

[bib66] Xu L, Meissner G (1998). Regulation of cardiac muscle Ca2+ release channel by sarcoplasmic reticulum lumenal Ca2+. Biophysical Journal.

[bib67] Xu L, Chirasani VR, Carter JS, Pasek DA, Dokholyan NV, Yamaguchi N, Meissner G (2018). Ca(2+)-mediated activation of the skeletal-muscle ryanodine receptor ion channel. The Journal of Biological Chemistry.

[bib68] Xu L, Harms FL, Chirasani VR, Pasek DA, Kortüm F, Meinecke P, Dokholyan NV, Kutsche K, Meissner G (2020). Single-channel properties of skeletal muscle ryanodine receptor pore Δ(4923)FF(4924) in two brothers with a lethal form of fetal akinesia. Cell Calcium.

[bib69] Yamaguchi N (2020). Molecular Insights into Calcium Dependent Regulation of Ryanodine Receptor Calcium Release Channels. Advances in Experimental Medicine and Biology.

[bib70] Zhang K (2016). Gctf: Real-time CTF determination and correction. Journal of Structural Biology.

[bib71] Zheng SQ, Palovcak E, Armache JP, Verba KA, Cheng Y, Agard DA (2017). MotionCor2: anisotropic correction of beam-induced motion for improved cryo-electron microscopy. Nature Methods.

[bib72] Ziman AP, Ward CW, Rodney GG, Lederer WJ, Bloch RJ (2010). Quantitative measurement of Ca2(+) in the sarcoplasmic reticulum lumen of mammalian skeletal muscle. Biophysical Journal.

