# Supplementary material for: Ca2+ inactivation of the mammalian ryanodine receptor type 1 in a lipidic environment revealed by cryo-EM
Source: eLife. 2022 Mar 8;11:e75568. doi: 10.7554/eLife.75568 (PMC8947763; doi:10.7554/eLife.75568)
Supplement: Transparent reporting form [file elife-75568-transrepform1.pdf]

eLIFE
1st Floor
24 Hills Road
Cambridge CB2 1JP, UK
P 01223 855340
W elifesciences.org
T @elife

eLife's transparent reporting form

We encourage authors to provide detailed information *within their submission* to facilitate the interpretation and replication of experiments. Authors can upload supporting documentation to indicate the use of appropriate reporting guidelines for health-related research (see EQUATOR Network), life science research (see the BioSharing Information Resource), or the ARRIVE guidelines for reporting work involving animal research. Where applicable, authors should refer to any relevant reporting standards documents in this form.

If you have any questions, please consult our Journal Policies and/or contact us: editorial@elifesciences.org.

Sample-size estimation

- You should state whether an appropriate sample size was computed when the study was being designed
- You should state the statistical method of sample size computation and any required assumptions
- If no explicit power analysis was used, you should describe how you decided what sample (replicate) size (number) to use

Please outline where this information can be found within the submission (e.g., sections or figure legends), or explain why this information doesn't apply to your submission:

To validate the new inactivated conformation, cryo-EM of RyR1-ACP/Ca^{2+} inactivated condition was carried out in duplicate as described in Results, Methods, Table S1. Duplicates are shown in paired figures Figs. 1-S5, Figs. S1-S3, Fig. S7a to demonstrate reproducibility.

For the tritiated ryanodine binding assay, a sample size of 4 was used.

For cryo-EM, the number of particles and the resolution attained are described in detail in Table S2.

Replicates

- You should report how often each experiment was performed
- You should include a definition of biological versus technical replication
- The data obtained should be provided and sufficient information should be provided to indicate the number of independent biological and/or technical replicates
- If you encountered any outliers, you should describe how these were handled
- Criteria for exclusion/inclusion of data should be clearly stated
- High-throughput sequence data should be uploaded before submission, with a private link for reviewers provided (these are available from both GEO and ArrayExpress)

Please outline where this information can be found within the submission (e.g., sections or figure legends), or explain why this information doesn't apply to your submission:

Independent assays, representing four distinct samples from an identical lot of total membrane fraction (purified from rabbit muscle) were used for the tritiated Ryanodine binding experiment. No ryanodine binding data was excluded.

One cryo-EM dataset of RyR1-ACP/EGTA was collected. Two independent cryo-EM datasets (A and B) of RyR1-ACP/Ca2+ were collected from two grids made from a single RyR1 purification batch as mentioned in Methods and Results. The high structural correlation between the inactivated model A and B (RMSD =0.95) in the central region of the channel is mentioned in results. During image processing, micrographs and particles were excluded following standard criteria as indicated in Methods and Figures S1, S3 and S4.

Statistical reporting

- Statistical analysis methods should be described and justified
- Raw data should be presented in figures whenever informative to do so (typically when N per group is less than 10)
- For each experiment, you should identify the statistical tests used, exact values of N, definitions of center, methods of multiple test correction, and dispersion and precision measures (e.g., mean, median, SD, SEM, confidence intervals; and, for the major substantive results, a measure of effect size (e.g., Pearson's r, Cohen's d)
- Report exact p-values wherever possible alongside the summary statistics and 95% confidence intervals. These should be reported for all key questions and not only when the p-value is less than 0.05.

Please outline where this information can be found within the submission (e.g., sections or figure legends), or explain why this information doesn't apply to your submission:

Methods: Resolution was measured using the FSC with 0.143 cutoff criterion

Fig. 1: Mean ryanodine binding from four replicas is shown in figure 1a with SEM error bars

Fig. 2: Inter-domain RMSD between different conformations

Figs. S1, S2, S3, Tables S1 and S2 contain all details of image processing and model building

Fig. S7: RMSD between two independent datasets for the relevant region of interest is 0.95 Å over 672 residues

Fig. S8: RMSD for the Ca2+ and ACP ligand is shown

RMSD for lipids and other residues of interest can be found in the Results section

(For large datasets, or papers with a very large number of statistical tests, you may upload a single table file with tests, Ns, etc., with reference to sections in the manuscript.)

Group allocation

eLIFE
1st Floor
24 Hills Road
Cambridge CB2 1JP, UK
P 01223 855340
W elifesciences.org
T @elife

- Indicate how samples were allocated into experimental groups (in the case of clinical studies, please specify allocation to treatment method); if randomization was used, please also state if restricted randomization was applied
- Indicate if masking was used during group allocation, data collection and/or data analysis

Please outline where this information can be found within the submission (e.g., sections or figure legends), or explain why this information doesn't apply to your submission:

N/A

Additional data files ("source data")

- We encourage you to upload relevant additional data files, such as numerical data that are represented as a graph in a figure, or as a summary table
- Where provided, these should be in the most useful format, and they can be uploaded as "Source data" files linked to a main figure or table
- Include model definition files including the full list of parameters used
- Include code used for data analysis (e.g., R, MatLab)
- Avoid stating that data files are "available upon request"

Please indicate the figures or tables for which source data files have been provided:

Details of the cryo-EM image processing and model validation are provided in Methods and figures S1, S3, S4, tables S1 and S2 in the supporting data file.
The cryo-EM map and models are available in the EMDB and PDB databases.

eLife Sciences Publications, Ltd is a limited liability non-profit non-stock corporation incorporated in the State of Delaware, USA, with company number 5030732, and is registered in the UK with company number FC030576 and branch number BR015634 at the address 1st Floor, 24 Hills Road, Cambridge CB2 1JP | August 2014
